# Synthesis and Photophysical Study of [(C^N^C)Au(L)]^n+^ Pincer Complexes, L = Alkynyl (n = 0), Isocyanide (n = +1): Comparative Analysis of Their Aggregation Behavior

**DOI:** 10.3390/molecules31193414

**Published:** 2026-09-25

**Authors:** Elizaveta V. Durova, Arina S. Stroeva, Kristina S. Kisel, Mikhail A. Kinzhalov, Viktor V. Sokolov, Georgiy A. Shonin, Dmitrii M. Nikolaev, Mikhail N. Ryazantsev, Sergey P. Tunik

**Affiliations:** Institute of Chemistry, Saint-Petersburg State University, Universitetskii pr., 26, 198504 Saint-Petersburg, Russia; st068755@student.spbu.ru (E.V.D.); st117556@student.spbu.ru (A.S.S.); k.kisel@spbu.ru (K.S.K.); m.kinzhalov@spbu.ru (M.A.K.); v.sokolov@spbu.ru (V.V.S.); st128251@student.spbu.ru (G.A.S.); d.nikolaev@spbu.ru (D.M.N.); m.ryazantsev@spbu.ru (M.N.R.)

**Keywords:** Au(III) pincer complexes, alkynyl and isocyanide ligands, aggregation induced emission, DFT calculations

## Abstract

Two series of Au(III) pincer complexes [(C^N^C)AuL]^n+^ (C^N^C = 2,6-diphenylpyridine, L = alkynyl, n = 0; L = isocyanide, n = 1) have been synthesized and characterized in solution by NMR spectroscopy and mass spectrometry. The solid-state structures of alkynyl complexes were also analyzed with XRD crystallography. In a dilute CH_2_Cl_2_ solution, both alkynyl and isocyanide complexes display a similar weak blue phosphorescence originating from a metal-perturbed ^3^ILCT state localized on the pincer ligand, but their solid-state emission characteristics differ significantly. The isocyanide complexes exhibit strong bathochromic shifts of emission band maxima compared to those observed in solution, which can be assigned to the formation of aggregates that afford excimers with short Au-Au contacts in the T_1_ emissive state. Among the neutral alkynyl analogues, two compounds retain essentially solution-like emissions, since their rigid crystalline environment precludes the excited-state transformation required for metal–metal bonding. Only the electron-rich dimethylamino derivative, which crystallizes as loosely packed dimers, exhibits a red-shifted solid-state band, and even this shift is substantially smaller than that of its isocyanide congener. The electronic structure of the complexes has been analyzed using DFT calculations, which corroborate the proposed rationalization of the observed trends in the emission characteristics and the difference in the aggregation behavior between the alkynyl and isocyanide chromophores.

## 1. Introduction

Cyclometalated Au(III) complexes supported by tridentate dianionic ligands (e.g., 2,6-diarylpyridines) constitute a promising class of luminescent gold chromophores showing rich photophysics and practically useful physico-chemical properties that pave the way to applications in a wide range of modern technologies, in particular, for the preparation of OLED devices with tunable emission energy [1,2,3,4,5]. Compared to complexes with mono- and bidentate ligands, these systems offer improved thermal stability, enhanced resistance to reduction to Au(I) species, and greater structural rigidity, the latter being of particular importance for emission efficacy, as it minimizes nonradiative excited-state relaxation.

An important option for the optimization of emission quantum yield for this type of Au(III) emitters is the introduction of strong-field ancillary ligands into a coordination sphere that decreases the accessibility of triplet d-d ligand field excited states and suppresses nonradiative d-d transitions that are usually a main channel of emission quenching. In this respect, a breakthrough was described in a seminal publication from Yam and coworkers [6], which demonstrated a synthetic approach to the preparation of the [(C^N^C)AuL] complexes containing strong σ-donating alkynyl ligands and exhibiting room temperature luminescence in solution. Later, the studies of this and many other research teams contributed strongly to the development of this chemistry, which also includes various C^N^C [7,8,9,10] and other tridentate (C^C^N [8,11,12,13], N^N^C [8], N^N^N [11,14]) ligands together with different ancillary ligands such as aryls, alkyls, N-heterocyclic carbenes, and phosphines, which have finally been summarized in a comprehensive review [15]. These findings stimulated development of the synthesis of this class of Au(III) complexes, the studies of their photophysics and applications in different areas, which is well documented in a series of review articles published in the last decade [1,2,3,4,15].

The square-planar geometry of Au(III) complexes, similar to the other d^8^ compounds (Pt(II), Pd(II), Rh(I)), makes possible the aggregation of Au(III) emitter molecules in a solid state and to produce (even in solution) the chromophores with modified photophysical characteristics (excited state character, emission energy and lifetime) [8,16,17,18,19,20]. In Au(III) aggregates, however, the higher formal charge of the metal center compared with the isoelectronic d^8^ analogues mentioned above brings about markedly different structural and photophysical features: metallophilic Au(III)···Au(III) interactions are much weaker so that aggregation is dominated by π–π and charge-transfer stacking rather than by metal–metal bonding. It is worth noting at this point that in many aspects, including aggregation phenomena, the alkynyl Au(III) complexes received much more attention compared to the other [(C^N^C)AuL] compounds, in particular, the complexes containing isocyanide ancillary ligands are one of the underexplored group of Au(III) chromophores. The paucity of photophysical studies on (C^N^C)Au(III) isocyanides seems to be largely a practical, rather than a fundamental issue. The cationic compounds are prone to oiling out and are frequently obtained as microcrystalline or amorphous solids, which commonly prevents obtaining diffraction-quality crystals and XRD structural study. Nevertheless, isocyanide complexes are of particular interest because they are structurally very similar to the alkynyl analogues but differ from them in the net charge of the molecule—a difference that, in combination with the high charge density of the Au(III) ion, could substantially affect aggregation behavior and the associated photophysical characteristics, as has been found in the present study. (See Scheme 1, where cationic isocyanide complexes exhibit higher trend to aggregation compared to neutral alkynyl relatives.)

In the present communication, we describe the preparation of two series of alkynyl and isocyanide complexes ([(C^N^C)AuL], L = alkynyl and isocyanide, see Scheme 2) that are essentially similar in their structure and steric characteristics but differ in the net charge of the molecules. The photophysics of the prepared chromophores have been studied in CH_2_Cl_2_ solution and in solid state. The observed differences in aggregation behavior are discussed and rationalized with the aid of DFT calculations, which provide insight into the structural reorganization of the aggregates in the excited state.

## 2. Results and Discussion

The general synthetic procedure for preparation of the target products is shown in Scheme 2.

The cyclometalated chlorogold precursors [Au(C^N^C)Cl] were prepared according to a procedure described in the literature [21]. The target cationic isocyanide and neutral alkynyl Au(III) complexes were subsequently synthesized from the chloride precursors following the reported protocols [16,22]. The complexes were purified by recrystallization via vapor diffusion of diethyl ether into concentrated acetonitrile or acetone solutions of the isocyanide and alkynyl complexes, respectively. All obtained complexes are new except for **Au-CC-anis**, which had been reported previously [16] and was used as a reference.

In solution, the obtained complexes were characterized by NMR spectroscopy, HR-ESI^+^, and MALDI mass spectrometry, see Figures S1–S14. All mass spectra displayed dominant signals corresponding to the molecular ions with isotopic distributions matching the calculated patterns. In HR-ESI^+^ mass spectra, the isocyanide complexes give a single dominant peak assigned to the intact [(C^N^C)Au(CNR)]^+^ cation, whereas the neutral alkynyls are detected as [M + H_3_O]^+^/[M + Na]^+^/adducts. Signal assignments in the ^1^H NMR spectra were carried out using the ^1^H-^1^H COSY data. Overall, the number of signals, their splitting patterns, and the integral ratios are fully consistent with the proposed compositions and molecular structures of the complexes shown in Scheme 2.

The alkynyl complexes produced single crystals suitable for X-ray diffraction studies and their solid-state structures were established using XRD analysis. The molecular views of the complexes are shown in Figure 1A. Crystallographic data, selected bond lengths, and angles are summarized in Tables S1 and S2. The obtained structures are typical for Au(III) complexes bearing pincer ligands. Three coordination sites around the gold center are occupied by the C^N^C functions, and the alkynyl ligand was found in the fourth position of coordination quadrangle.

The key bond lengths and angles of the obtained complexes are not exceptional and fall in the range typical for the reported related structures [6,16,19,23,24,25]. The fragments of the complexes’ molecular packing are shown in Figure 1B. The molecules of **Au-CC-styr** and **Au-CC-anis** form infinite chains in which the molecules are rotated relative to each other by 136.6° and 128.6°, respectively, whereas the **Au-CC-dmal** molecules form isolated dimers with head-to-tail orientation. None of the packing patterns displays short Au···Au contacts. The interaction of adjacent molecules can be described in terms of π-stacking between aromatic systems of the pincer ligands with the interplane distances in the range of 3.28–3.36 Å. The structure of the **Au-CC-anis** was recently reported [16] and the major structural parameters are essentially similar to those obtained in the present study. Slight deviations are evidently due to the different crystallization conditions.

Thus, six Au(III) complexes were successfully synthesized and fully characterized in solution, with the HR-ESI^+^ and MALDI mass-spectrometry and NMR spectroscopy data confirming their proposed compositions and molecular structures. X-ray diffraction analysis of the alkynyl complexes revealed the expected square-planar coordination geometry, while their crystal packing is governed by rather weak π-π interactions and does not display short Au-Au contacts. Unfortunately, none of the isocyanide complexes yielded single crystals suitable for XRD analysis. Therefore, the intermolecular interactions between these chromophores in solid state and the resulting variations in their photophysical parameters were analyzed by using DFT and DFT calculations for the aggregates that can be formed upon packing the complexes’ molecules in a crystal cell.

## 3. Photophysical Properties

The photophysical properties of the obtained complexes were studied in dichloromethane solution and in solid phase. The absorption, excitation, and emission spectra are shown in Figure S16, Figure 2 and Figure 4. The photophysical data are summarized in Table 1.

The UV-vis absorption spectra of the alkynyl and isocyanide complexes in CH_2_Cl_2_ display similar patterns consisting of intense high-energy bands below 330 nm and less intense low-energy bands or shoulders in the 360–430 nm region, see Figures S16–S22. The high-energy bands can be assigned to intraligand ^1^LC transitions [7]. The lower-energy bands can be described in terms of metal-perturbed intraligand charge-transfer (^1^ILCT) transition [7,11]. The assignment of low energy absorption to metal-to-ligand charge-transfer ^1^MLCT transitions is unlikely, owing to the electrophilic and redox-inactive character of the Au(III) center. At the same time, **Au-CC-dmal** and **Au-CN-dmal** may also include an additional ligand-to-ligand (from alkynyl/isocyanide to pincer ligand) charge-transfer contribution (^1^LLCT) in the low-energy area of the spectrum [7] due to the electron-rich nature of the dimethylamino substituents.

In diluted CH2Cl2 solution (10^−5^ mol/L), the alkynyl complexes display a weak blue phosphorescence, see Figure 2. The emission spectra of **Au-CC-styr** and **Au-CC-dmal** show structureless bands, whereas **Au-CC-anis** displays vibronically structured emission band with maxima at 474 and 506 nm, which fits well with the vibronic progression of ca. 1350 nm typical for aromatic systems involved in the formation of an emissive excited state. The character of emissive excited states for the alkynyl complexes may be described as a mixture of metal-perturbed intraligand charge transfer (^3^ILCT) evidently localized on the C^N^C ligand and ligand to metal charge transfer (^3^LMCT) with different contributions of the components. It is worth noting that **Au-CC-styr** does not display a concentration dependence of emission that indicates the absence of its aggregation in the concentration range 10^−5^–10^−3^ mol/L, Figure S17. **Au-CC-anis** shows only a slight red shift of its emission band upon a concentration increase to 10^−3^ mol/L (Figure S18), which suggests a weak aggregation or stronger contribution of ^3^LMCT character. On the contrary, in diluted solution of **Au-CC-dmal**, one can observe a novel weak band red shifted to 650 nm (Figure 2) which becomes the dominating emission feature at higher (10^−3^ mol/L) concentration. (See Figure S19). In this case, the aggregation-induced red shift (compared to the major emission band in diluted solution) implies a change in the nature of the excited state from ^3^ILCT to the charge transfer from ligand orbitals to MM bond (^3^LMMCT), *vide infra*. The emission character transformation is accompanied by a substantial decrease in the energy gap between the ground and emissive states, thus shifting the emission into the deep-red region of the spectrum. The stronger aggregation effect observed for **Au-CC-dmal** can be rationalized by the higher contribution of electrostatic (dipole–dipole) noncovalent interactions arising from the polarization of the molecule that contains a positively charged metal ion and an electron-rich amino group in the alkynyl ligand, which plays a key role in intermolecular attraction.

In the solid state, **Au-CC-styr** and **Au-CC-anis** display emission band profiles that closely resemble those observed in solution, with a slight increase in intensity of the long wavelength tails (Figure 2B). This observation is fully consistent with the crystal cell packing shown in Figure 1 that display a very weak π-stacking and absence of short Au-Au contacts. In this context, the molecules in the crystal lattice can be considered as essentially isolated chromophores, incapable of forming AIE aggregates. The photophysical behavior of **Au-CC-anis** has been studied earlier [16]. The data obtained in the present study are in general agreement with those reported in the abovementioned publication excluding values of quantum yields and excited state lifetimes in solution. The latter was only qualitatively estimated [16] and the difference in the quantum yields magnitudes is not critical, taking into account a very weak emission of the chromophore. The solid-state spectrum reported previously [16] shows a broad featureless emission with the maximum at 555 nm, whereas the **Au-CC-anis** sample (Figure 2B) displays a structured band with the components at 472, 503, 554, and 599 nm, which fits well beneath the unstructured profile obtained in the previous experiment [16]. Considering that the crystal structures are nearly identical, we attribute the previously observed emission band broadening to partial destruction of the crystals during sample preparation for the photophysical experiments, which smeared out the well-resolved vibronic structure associated with the C^N^C-centered emissive excited state.

In contrast, in the solid state **Au-CC-dmal** displays a broadened emission band shifted to 555 nm (see Figure 2B) that points to the difference in the behavior of the **Au-CC-dmal** dimers compared to the other alkynyl complexes. The emission band red shift is evidently dictated by a reorganization of the dimeric aggregates in excited state to give structural pattern favorable for manifestation of AIE features. The difference between **Au-CC-dmal** and two other compounds is determined by the mode of molecular packing in crystal cells. **Au-CC-styr** and **Au-CC-anis** shows rather rigid columnar structure whereas **Au-CC-dmal** molecules form isolated dimeric aggregates that are relatively loosely packed (Figure 1B), which allows structural transformation of the aggregates upon excited state relaxation. It is interesting that the emission band of the aggregates formed in solution (Figure S19) is further red shifted up to 640 nm. That is indicative of another structure of the aggregates with more effective interaction of the chromophores. It is worth noting at this point that the a configuration of the dimers in crystal cell does not allow unrestricted transformation due to environmental constraints, whereas in solution interacting molecules are evidently free in selection of aggregation modes both in the ground and the excited state, which allows a choice of the optimal aggregate configuration and stronger aggregation effects.

DFT analysis of the **Au-CC-dmal** dimers’ electronic structure is in agreement with the observed trends in the variations of emission maxima for the aggregates formed in solution and in solid state. Indeed, we found two minima on the potential surface of the dimers in the ground state, which exhibited formation of excited state triplets (T_1_) with a substantially different S_0_-T_1_ energy gap, Figure 3, Figures S23 and S24. One of the structures is essentially similar to that observed in the solid state, with head to tail molecular packing and Au-Au contacts substantially longer than the sum of van der Waals radii. Transition to the T_1_ state results in contraction of the Au-Au distance, giving an emission wavelength red-shifted by ca. 70 nm relative to the value calculated for an isolated chromophore. The other ground state structural pattern displays a twist of 90° in the orientation of the molecules, a shorter Au-Au distance, and intermolecular π-stacking between aromatic systems of pincer ligands and between the pincer ligand and CN triple bond. In the excited T_1_ state, this dimer keeps the mutual orientation of the molecules nearly the same, but the Au-Au distance has been shortened, creating a further shift of calculated emission maximum of ca. 30 nm. The optimized structures may be assigned to the aggregates formed in crystal lattice and in solution, respectively, taking into account the similarity of the first dimer structure with that found in solid state and the possible flexibility of intermolecular interaction in solution. It should be noted that the results of our calculations do not completely fit the experimental emission band maxima, but they do reproduce well the observed trend in variations of the S_0_-T_1_ energy gap in solid state and in solution.

Similar to the alkynyl complexes in diluted CH_2_Cl_2_ solution, the isocyanide complexes display a very weak phosphorescence with emission band maxima at 468, 433 and 437 nm (Figure 4, Table 1) for **Au-CN-styr**, **Au-CN-anis**, and **Au-CN-dmal**, respectively, and excited-state lifetimes from 22 to 44 ns. The lifetime values of the order of tens of nanoseconds may be explained by fast nonradiative excited state relaxation that also results in low emission quantum yields, which however show an increase upon deaeration of the solution from 0.4 to 0.9% for **Au-CN-anis** and from 0.2 to 0.3% for **Au-CN-dmal**. For **Au-CN-styr** and **Au-CN-anis**, the emission character may be assigned to metal-perturbed intraligand (C^N^C) charge transfer, ^3^ILCT, with negligible participation of the isocyanide ligand. This is consistent with the literature data [7,11] and results of quantum chemical calculations (Figure 5 and Tables S7 and S8). In contrast, for **Au-CN-dmal**, the presence of the dimethylamino group on the isocyanide ligand significantly modifies the character of the emissive state. Electron-density transfer from the NMe_2_ substituent plays a dominant role in the emissive transition (Figure 5 and Table S9).

In the solid state, the isocyanide complexes display strong red shifts of emission bands relative to a dilute solution. The shift amounts to 158, 202, and 235 nm for **Au-CN-styr**, **Au-CN-anis**, and **Au-CN-dmal**, respectively, see Figure 4B and Table 1. The observed bathochromic shift suggests the formation of lower energy emissive excited states, which may be associated with the chromophore’s aggregation involving π-π interactions and formation of Au-Au contacts, which usually result in the excited state character transformation to charge transfer from ligands to metal-metal bond (^3^LMMCT) [8]. However, for the isocyanide complexes under study, the calculations under the framework of the aggregated dimers model (Figure S25 and Tables S10–S12) did not give energy gap values even when approaching the experimental data. The discrepancy is itself informative: it indicates that the emissive species cannot be a discrete pair and that the exciton is delocalized over a longer stack. It has been earlier shown experimentally and theoretically [26] that an increase in the number of the square planar Pt(II) chromophores taking part in the aggregation results in a substantial reduction of the T_1_-S_0_ energy gap, provided that the packing of the molecules does allow this type of aggregation. Therefore, the columnar head-to-tail (or slightly skewed) arrays accompanied by short Au-Au contacts represent a well-documented motif for the C^N^C Au(III) complexes [16]. Consequently, the trimer represents the minimal model capable of capturing the cooperative nature of interaction in the aggregate, and we adopt it as a working hypothesis for the exciton delocalization length. It is also worth noting that aggregated species of higher nuclearity, e.g., tetrameric aggregates, indeed display further red shifts of emission band (see Table S16) that indicate the role of exciton delocalization in the aggregates. The general trend is thus evident, but computational difficulties increase exponentially, which prevented analysis of higher aggregates.

Ground-state optimization of the trimers, Figure S26, converges to skewed stacks with two inequivalent Au•••Au separations, neither of which is shorter than the sum of the gold van der Waals radii. However, optimized structures of excited T_1_ states display the trimers with the ordered head-to-tail orientation and short Au-Au contacts in the 3.30–3.32 Å range that is indicative of effective metallophilic interaction. The calculated emission wavelengths for these relaxed excited-state aggregates reproduce well the experimental solid-state maxima (Figure 5), and correctly rank the three compounds.

Natural transition orbitals (NTO) diagram (Figure 5) together with interfragment charge transfer (IFCT) decomposition (Tables S13–S15) indicate major contribution of intermolecular ligand to ligand (LLCT) and ligand to metal (LMMCT) charge transfer processes responsible for the formation of emissive excited states. The contribution of the third chromophore to the NTO does not appear dominative, but it proved to be critical for the T_1_ energy reduction and red shift of emission wavelengths. The role of the third molecule most probably consists of polarizing and rigidifying the aggregate rather than participating directly in the T_1_-S_0_ transition. The latter distinction rationalizes why the dimer model, although capturing the NTO character correctly, underestimates the energy gap reduction and fails quantitatively. The very low solid-state quantum yields (<0.1%) of the isocyanide complexes in solid state indicate that the considerable emission red shift is accompanied by efficient nonradiative excited state relaxation in accord with the EnergyGapLaw [27,28,29].

One of the aims of this study was to compare the aggregation behavior of isocyanide and alkynyl complexes under essentially similar conditions that imply nearly equal steric characteristics of the pairs of molecules, which determined the choice of the complexes for investigation. In line with the variations in the coordinating functionality (NC-R vs. CC-R), these types of complexes also exhibit different net charges (+1 vs. 0), which may also be essential for aggregation behavior. A comparison of emission spectra of alkynyl and isocyanide analogues, Figure 6, indicates that for the alkynyl complexes with styryl and anisyl substituents, emission wavelengths in the solid state are nearly identical to those observed in solution, whereas the analogous isocyanide chromophores display a strong aggregation effect characterized by the red shift of emission of ca. 150 and 200 nm, respectively. For the dimethylamino pair, the alkynyl complex does aggregate, but the shift (129 nm) remains well below that of its isocyanide counterpart (235 nm).

These observations appear counterintuitive at first glance. Despite a positive net charge that should, in principle, disfavor chromophores’ contacts, the isocyanide complexes are prone to forming aggregated structures in the crystal lattice that exhibit typical AIE characteristics. This counterintuitive behavior may be explained by taking into account a few important features that differentiate the alkynyl and isocyanide complexes. First, the π-accepting nature of the isocyanide fragment stimulates its noncovalent interaction with the π-donor aromatic system of the pincer ligand that is clearly visible in the optimized structures of the ground and excited states of the isocyanide aggregates, Figures S26 and S27. This complementary donor–acceptor stacking further stabilizes the columnar arrangement of the cations.

Second, the isocyanide complexes are salts, and their crystal packing is expected to be governed by the segregation of cationic and anionic domains. This charge separation drives the [(C^N^C)Au(CNR)]^+^ cations into columnar stacks, segregated from the OTf^−^ counterions, a motif well documented for cationic square-planar d^8^ systems [16,26] and mirrored by the optimized aggregate structures obtained here. In such ionic lattices, the Coulombic repulsion between the cations is offset by the overall Madelung stabilization, so that columnar stacking is enforced rather than avoided. In contrast, the neutral alkynyl complexes are free to adopt the packing that optimizes dispersion interactions. As confirmed by XRD, this results in isolated dimers or loosely π-stacked chains, without any requirement for the collinearity of the Au centers.

Superimposed on these factors is the effect of the NMe_2_ substituent. The strongly electron-donating amine polarizes the molecule, creating a ground-state dipole that favors head-to-tail association. Accordingly, **Au-CC-dmal** is the only alkynyl complex of the series that crystallizes as discrete dimers and the only one that displays a solid-state red shift. The same substituent effect also promotes its aggregation in concentrated solution. Notably, the aggregation patterns differ in the two media: the emission of the aggregates formed in solution (640 nm) is red-shifted relative to that of the crystalline sample (555 nm), demonstrating that environmental constraints in the latter case prevent the aggregate from attaining the most favorable excited-state configuration, whereas in solution such constraints are much less restrictive.

## 4. Conclusions

Two series of Au(III) complexes based on 2,6-diphenylpyridine as tridentate C^N^C ligand and containing alkynyl/isocyanide ligands in the fourth position of the coordination quadrangle have been synthesized and characterized using mass-spectrometry and NMR spectroscopy. The solid-state structures of the alkynyl complexes were determined with XRD crystallography, where the crystal packing patterns revealed a weak intermolecular π-stacking between aromatic systems of the pincer ligands with no ground-state short Au-Au contacts. In a diluted CH_2_Cl_2_ solution, both types of the complexes emit weakly in the blue from a metal-perturbed ^3^ILCT state localized on the C^N^C ligand, whereas in solid state photophysical behavior of the alkynyl and isocyanide complexes is substantially different. The neutral **Au-CC-styr** and **Au-CC-anis** complexes exhibit emission bands that are essentially similar to those observed in solution, in contrast to all three cationic isocyanide analogues, which show a strong red shift of emission in solid state due to multiple interaction between mononuclear chromophores in the triplet excited state. The presence of the electron-donating NMe_2_ substituents in the alkynyl and isocyanide ligands of the **Au-CC-dmal** and **Au-CN-dmal** complexes polarizes the corresponding molecules, which enhances their propensity to aggregate in the crystal lattice. This effect is substantially stronger for the isocyanide counterparts. The observed general trend of the charged isocyanide complexes to exhibit higher aggregation propensity looks somewhat counterintuitive, but it can be rationalized by taking into account π-stacking of the pincer and isocyanide ligands and stronger dipole-dipole interaction in the case of the isocyanide molecules bearing positive net charge, which compensates the electrostatic repulsion forces.

## 5. Experimental Section

### 5.1. Materials

General and deuterated solvents were used as received, excluding acetonitrile and dichloromethane. Acetonitrile was boiled over P_2_O_5_ (20 g/L) for 4 h followed by distillation at atmospheric pressure. Dichloromethane was boiled over P_2_O_5_ (20 g/L) for 3 h and then distilled at atmospheric pressure.

Commercially available precursors were used in the synthesis of target compounds: KAuCl_4_ (98%, Sigma Aldrich, Steinheim am Albuch, Germany), 2,6-diphenylpyridine (98%, BLDpharm, Shanghai, China), 1-ethynyl-4-methoxybenzene (99%, Alpha Aesar, Lancashire, UK), 2,6-di-p-tolylpyridine (97%, BLDpharm, Shanghai, China), triethylamine hydrochloric acid (98%, Vecton, St. Petersburg, Russia), silver(I) triflate (98%, Alfa Aesar, Lancashire, UK), copper(I) iodide (98%, Sigma Aldrich, Gillingham, UK).

1-ethynyl-4-vinylbenzene was synthesized according to the published procedures [30]. 4-Isocyano-N,N-dimethylaniline and 1-isocyano-4-methoxybenzene were synthesized according to a modified literature procedure [31], while 1-isocyano-4-vinylbenzene was prepared following the reported method [22].

4-Ethynyl-N,N-dimethylaniline was prepared from aniline in four steps. N,N-Dimethylaniline, 4-iodo-N,N-dimethylaniline, and N,N-dimethyl-4-[(trimethylsilyl)ethynyl]aniline were synthesized according to reported procedures in 85, 89, and 92% yield, respectively [32,33,34]. The TMS-protected intermediate (1 equiv) was dissolved in DMF/H_2_O (10:1) and treated with KF (1.5 equiv) at room temperature for 1 h. The reaction mixture was diluted with saturated aqueous solution of NH_4_Cl and extracted with EtOAc. The organic phase was dried over Na_2_SO_4_, concentrated, and the residue was purified by column chromatography (hexane/EtOAc, 20:1) to afford the target compound as a pale oil in 91% yield.

^1^H NMR (400 MHz, Chloroform-*d*) δ 7.39 (d, *J* = 8.9 Hz, 2H), 6.64 (d, *J* = 8.9 Hz, 2H), 3.00 (s, 7H).

### 5.2. General Experimental Details

The NMR spectra were recorded on a Bruker Avance III 400 MHz NMR spectrometer (9.4 T, 400 MHz; Ettlingen, Germany). Residual proton resonances from deuterated solvents were used to reference the ^1^H NMR spectra. High-resolution electrospray ionization mass spectra were recorded in the positive-ion mode on a Bruker Daltonics micrOTOF series instrument (Bremen, Germany). MALDI mass spectra were recorded on a Bruker UltrafleXtreme MALDI-TOF/TOF mass (Bremen, Germany). Additional high-resolution electrospray ionization mass spectra were acquired in the positive-ion mode on a Q Exactive Plus hybrid quadrupole-Orbitrap mass spectrometer (Thermo Fisher Scientific, Bremen, Germany) equipped with an atmospheric-pressure electrospray ionization source. Data acquisition and primary processing for the Brucker and Q Exactive Plus measurements were performed using Bruker compass dataAnalysis 4.0 and Thermo Fisher Scientific Xcalibur 2.8 softwares respectively. The obtained mass spectra were further analyzed and interpreted using mMass software 5.5.0.

In the synthesis of the isocyanide complexes and during the photophysical measurements, the solutions were degassed by bubbling argon for 15 min. The recrystallization of these complexes was carried out using carefully dried solvents.

### 5.3. General Procedure for the Synthesis

#### 5.3.1. Au(CNC)Cl Chloride Complexes Synthesis

The chloride complexes synthesis was carried according to slightly modified published procedure [21]. 2.16 mmol of C^N^C ligand precursor (2,6-diphenylpyridine) was mixed with 4.32 mmol of mercury(II) acetate and dissolved in 25 mL of absolute ethanol. The mixture was degassed and then heated at 100 °C for 1.5–2 h under vigorous stirring. 7.57 mmol of lithium chloride were dissolved in 15 mL of methanol and added to the cooled reaction mixture. The mixture was then heated to 60 °C and stirred for 30–60 min. Finally, 60–100 mL of distilled water was added and a precipitate of the product was immediately formed. The precipitate was filtered and washed with distilled water and cold methanol successively. The yellowish amorphous product Hg(C^N^C)Cl was used in the next step without further purification.

0.43 mmol of Hg(C^N^C)Cl and 0.43 mmol of KAuCl_4_ were boiled in 15 mL of acetonitrile at 80 °C for 24 h in inert atmosphere. After cooling to room temperature, the excess solvent was evaporated and the yellowish precipitate was filtered, washed with diethyl ether, and dried in air.

#### 5.3.2. Au(III) Isocyanide Complexes Synthesis

The isocyanide complexes **Au-CN-styr**, **Au-CN-anis**, and **Au-CN-dmal** were synthesized using a modified literature procedure [8]. 0.11 mmol of Au(C^N^C)Cl and 0.11 mmol of AgSO_3_CF_3_ were suspended in acetonitrile, and the resulting mixture was degassed and left at 80 °C for 1.5–2 h. After cooling, the mixture was filtered to remove AgCl, and the solvent was evaporated. Then the intermediate acetonitrile complex was redissolved in 20 mL of dichloromethane and 0.11 mmol of isocyanide ligand with a small excess was added. The reaction was conducted at 40 °C for 2 h. Finally, the product was purified by vapor diffusion recrystallization in an acetonitrile-diethyl ether inert system at 0 °C.

#### 5.3.3. Au(III) Alkynyl Complexes Synthesis

The alkynyl complexes **Au-CC-styr**, **Au-CC-anis** and **Au-CC-dmal** were synthesized using a modified literature procedure [8]. 0.11 mmol of alkynyl ligand with a small excess was added to 0.11 mmol of Au(C^N^C)Cl and dissolved in dichloromethane in the presence of triethylamine hydrochloric acid (0.49 mmol) and a catalyst (CuI). The suspension was stirred at 40 °C for 24 h. The resulting mixture was filtered and the product was recrystallized in an acetone-diethyl ether system at 5 °C.

**Au-CC-styr**. Yellow needles. 25 mg (Yield 32%). ^1^H NMR (400 MHz, acetonitrile-d_3_) δ 8.07 (d, *J* = 8.1 Hz, 1H), 8.03 (d, *J* = 7.1 Hz, 2H), 7.78 (d, *J* = 8.2 Hz, 2H), 7.74 (d, *J* = 8.1 Hz, 2H), 7.56 (d, *J* = 8.3 Hz, 2H), 7.49 (d, *J* = 8.5 Hz, 2H), 7.44 (d, *J* = 6.3 Hz, 2H), 7.35 (t, *J* = 7.0 Hz, 2H), 6.81 (dd, *J* = 17.7, 11.0 Hz, 1H), 5.87 (d, *J* = 17.9 Hz, 1H), 5.32 (d, *J* = 10.9 Hz, 1H). HR-ESI/MS (*m*/*z*): calcd. for C_27_H_19_AuN^+^ 554.1183 (found 554.1177).

**Au-CC-anis**. Bright orange prismatic crystals. (Yield 27%). ^1^H NMR (400 MHz, CD_2_Cl_2_) δ 8.10 (d, *J* = 7.3 Hz, 2H), 7.96 (t, *J* = 8.0, 8.0 Hz, 1H), 7.69 (d, *J* = 7.7 Hz, 2H), 7.60 (d, *J* = 8.0 Hz, 2H), 7.57 (d, *J* = 8.6 Hz, 2H), 7.46 (t, *J* = 7.4, 7.4 Hz, 2H), 7.34 (t, *J* = 7.4, 7.4 Hz, 2H), 6.93 (d, *J* = 8.6 Hz, 2H). HR-ESI/MS (*m*/*z*): calcd. for C_26_H_21_AuNO_2_^+^ 576.1232 (found 576.12360).

**Au-CC-dmal**. Bright orange prismatic crystals. (Yield 22%). ^1^H NMR (400 MHz, DMSO) δ 8.31 (s, 1H), 8.18 (t, *J* = 8.0, 8.0 Hz, 1H), 7.99 (d, *J* = 8.0 Hz, 2H), 7.97–7.89 (m, 4H), 7.48–7.41 (m, 2H), 7.40–7.31 (m, 4H), 6.72 (d, *J* = 8.7 Hz, 2H), 2.95 (s, 6H). HR-ESI/MS (*m*/*z*): calcd. for C_27_H_24_AuN_2_O^+^ 589.1549 (found 589.15485).

**Au-CN-styr**. Dark brown cotton-like precipitate. ^1^H NMR (400 MHz, Methylene Chloride-*d*_2_) δ 7.95 (t, *J* = 7.8 Hz, 1H), 7.72 (d, *J* = 7.2 Hz, 2H), 7.62 (d, *J* = 8.0 Hz, 2H), 7.52 (dd, *J* = 14.8, 8.1 Hz, 6H), 7.43 (t, *J* = 7.6 Hz, 2H), 7.34 (t, *J* = 7.3 Hz, 2H), 6.83–6.72 (m, 1H), 5.89 (d, *J* = 18.0 Hz, 1H), 5.45 (d, *J* = 11.5 Hz, 1H). HR-ESI/MS (*m*/*z*): calcd. for C_26_H_20_AuN_2_^+^ 557.1287 (found 557.12836).

**Au-CN-anis**. Green-yellow needle-shaped crystals. (Yield 28%). ^1^H NMR (400 MHz, DMSO) δ 8.14 (t, *J* = 8.0, 8.0 Hz, 1H), 7.99 (d, *J* = 8.0 Hz, 2H), 7.95 (s, 2H), 7.66–7.56 (m, 4H), 7.42–7.34 (m, 2H), 7.31 (td, *J* = 7.7, 7.6, 1.5 Hz, 2H), 6.90 (d, *J* = 8.9 Hz, 2H), 3.75 (s, 3H). MALDI-MS (*m*/*z*): calcd for C_25_H_18_AuN_2_O^+^ [M^+^] 559.1085, found 559.1068.

**Au-CN-dmal**. Bright orange cotton-like precipitate. (Yield 35%). ^1^H NMR (400 MHz, CD_2_Cl_2_) δ 8.14 (t, *J* = 8.1 Hz, 1H), 7.85 (dd, *J* = 7.2, 1.4 Hz, 2H), 7.76 (dd, *J* = 7.6, 1.6 Hz, 2H), 7.69 (dd, *J* = 8.6, 6.1 Hz, 4H), 7.54 (td, *J* = 7.4, 1.6 Hz, 2H), 7.51–7.44 (m, 2H), 6.88–6.79 (m, 2H), 3.18 (s, 6H). MALDI-MS (*m*/*z*): calcd for C_26_H_21_AuN_3_^+^ [M^+^] 572.1401, found 572.1328.

### 5.4. Photophysical Experiments

UV-Vis absorption spectra were recorded in dichloromethane solutions using a Shimadzu UV-1800 spectrophotometer (Shimadzu, Kyoto, Japan). Emission spectra were acquired on an Avantes AvaSpec-2048x64 spectrometer (Avantes, Apeldoorn, The Netherlands) under excitation at 365 nm using a LED source. Excitation spectra were measured with a Shimadzu UV-2550 spectrophotometer (Kyoto, Japan).

Emission quantum yields (Φ) were determined using the comparative method, with an aerated aqueous solution of Ru(bpy)_3_[PF_6_]_2_ as the reference (Φ_r_ = 0.042), according to the following equation:

$$\Phi_{s}=\Phi_{r}\frac{n_{s}^{2}A_{r}I_{s}}{n_{r}^{2}A_{s}I_{r}}$$

where Φ_s_ is the quantum yield of the sample, Φ_r_ is the quantum yield of the reference, n is the refractive index of the solvent (n(H_2_O) = 1.333, n(CH_2_Cl_2_) = 1.424), A is the absorbance (A_s_—of the samples, A_r_—of the reference) at the excitation (365 nm) wavelength, and I is the integrated emission intensity obtained from the respective emission bands.

For solid-state samples, quantum yields were measured using a Fluorolog-3 spectrofluorometer (HORIBA Jobin Yvon, Edison, NJ, USA) equipped with an integrating sphere (Quanta-phi, HORIBA Scientific, Edison, NJ, USA). Excitation spectra for these samples were recorded on the same instrument.

Excited-state lifetimes were determined using time-correlated single-photon counting (TCSPC). A pulsed DTL-375QT laser, Laser-Compact Group, Moscow, Russia (355 nm, 1 kHz repetition rate) served as the excitation source. The detection system comprised a Tektronix DPO2012B oscilloscope, Tektronix, Oregon, United States (100 MHz bandwidth), an Ocean Optics Monoscan-2000 monochromator, Ocean Optics, Florida, USA (1 nm resolution), a FASTComTec MCS6A1T4 time digitizer, (Oberhaching, Germany), and a Hamamatsu H10682-01 photomultiplier tube (Hamamatsu, Japan). Emission decay curves were processed using Origin 9.0 software and lifetime components were calculated via mono- or biexponential fitting.

### 5.5. Single-Crystal XRD Analysis

All the crystals were selected under a polarizing microscope, coated with cryoprotective oil, mounted on nylon loops, and measured at 100 K. X-ray diffraction data were collected at the Centre for X-ray Diffraction Studies of the Research Park of St. Petersburg State University using a Rigaku Oxford Diffraction XtaLAB SuperNova HyPix-3000 single-crystal diffractometer with Cu Kα radiation (λ = 1.54184 Å).

The diffraction data were processed with the CrysAlisPro software package, version 1.171.41.94a [35]. Structure solution was carried out by the dual-space algorithm implemented in SHELXT [36,37], and subsequent refinement was performed in the OLEX2 package [38]. All non-hydrogen atoms were refined anisotropically. Hydrogen atoms were introduced at geometrically calculated positions and refined using the ‘riding’ model, with C–H distances of 1.077–1.083 Å and $U_{iso}(H)$ fixed at 1.2–1.5 times the $U_{eq}$ value of the corresponding parent atom. The crystallographic data have been deposited with the Cambridge Crystallographic Data Centre and are available free of charge from the CCDC database www.ccdc.cam.ac.uk/structures/ (accessed on 2 August 2026). The unit-cell and refinement parameters are summarized in Table S1.

**Au-CC-styr**. C_29_H_22_AuNO_0.666667_, P-1 (no. 2), *a* = 9.46560(10) Å, *b* = 16.8984(2) Å, *c* = 21.2564(2) Å, *α* = 95.8790(10)°, *β* = 99.2390(10)°, *γ* = 90.0000(10)°, *V* = 3337.81(6) Å^3^, *Z* = 6, *R*_1_ = 0.0451, CCDC 2578199. **Au-CC-anis**. C_52.5_H_38_Au_2_ClN_2_O_2.5_, P-1, *a* = 8.9570(3) Å, *b* = 12.5989(4) Å, *c* = 19.0018(4) Å, *α* = 77.267(2)°, *β* = 78.172(2)°, *γ* = 78.402(3)°, *V* = 2019.71(11) Å^3^, *Z* = 2, *R*_1_ = 0.0539, CCDC 2578200. **Au-CC-dmal**. C_27_H_21_AuN_2_, P2_1_/c, *a* = 15.30460(10) Å, *b* = 11.78000(10) Å, *c* = 11.67660(10) Å, *β* = 103.8300(10)°, *V* = 2044.12(3) Å^3^, *Z* = 4, *R*_1_ = 0.0223, CCDC 2578201.

### 5.6. Computational Methods

All quantum-chemical calculations were carried out with the Gaussian 09 (Revision D.01) program package [39]. Ground-state (S_0_) minimum structures for all species were obtained by geometry optimization on the S_0_ potential energy surface. For the isocyanide complexes, calculations were carried out at the MN12SX/(6-31G*, Au SDD) level of theory, whereas for the alkynyl complexes the M062X/(6-31G*, Au SDD) level of theory was employed. Solvent effects were taken into account for the monomeric models of Au(III) complexes by using the Polarizable Continuum Model with dichloromethane (PCM = DCM) as the dielectric medium.

The triplet T_1_ minima were located via an excited-state relaxation protocol. First, we optimized the geometry on the S_1_ potential energy surface, starting from the Franck-Condon geometry and following the relaxation pathway into the region of S_1_/T_1_ intersystem crossing (ISC). The S_1_ forces along this trajectory were evaluated within the TD-DFT formalism. From structures in the ISC region, the nearby T_1_ minimum was then identified and optimized on the T_1_ surface. All calculations for triplet states were performed using an unrestricted approach. Emission band maxima were estimated as the vertical energy gap between T_1_ and S_0_, calculated at the T_1_ minimum geometry.

## Figures and Tables

**Scheme 1:**
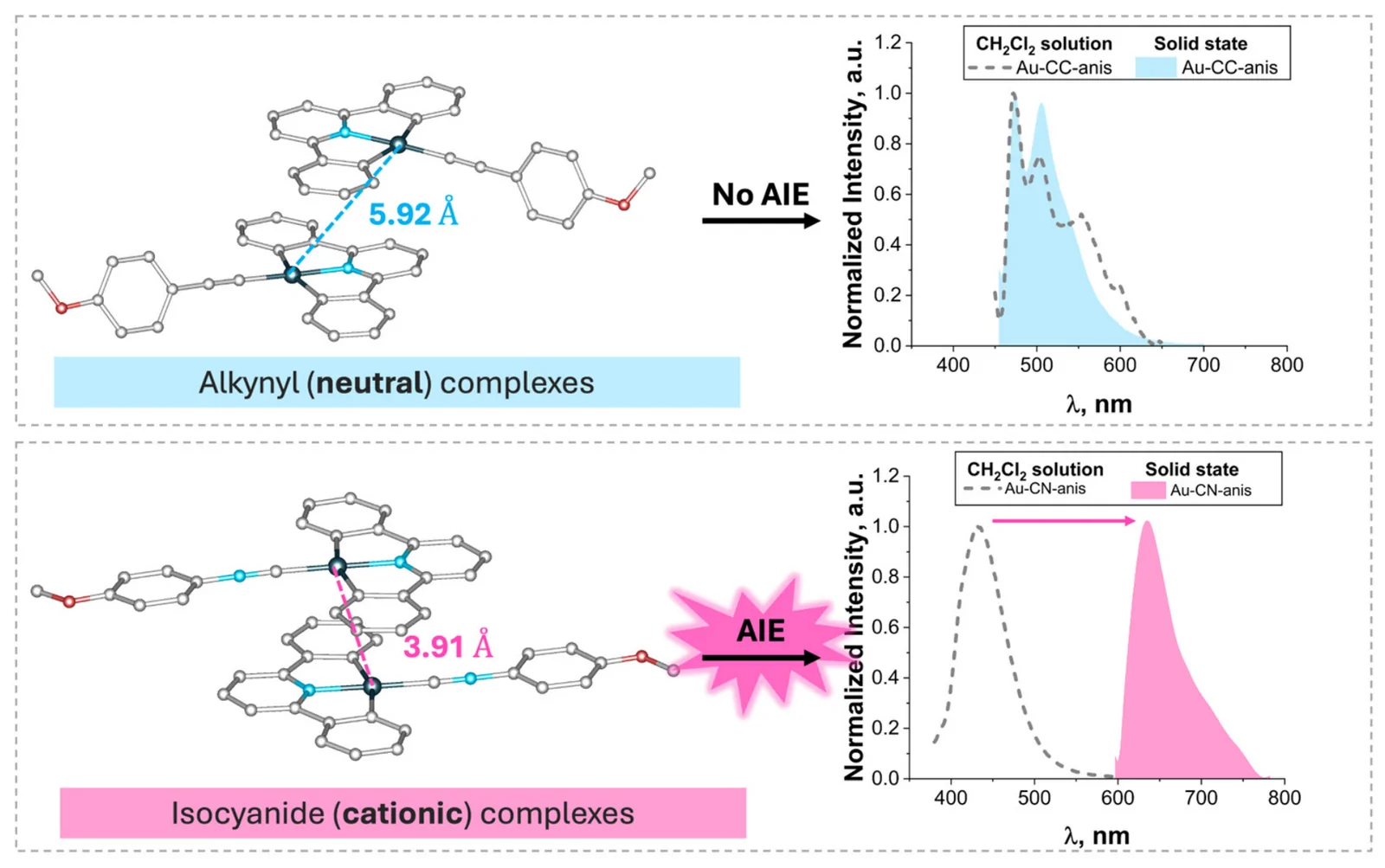
Counter-intuitive aggregation behavior of the pincer alkynyl and isocyanide emitters.

**Scheme 2:**
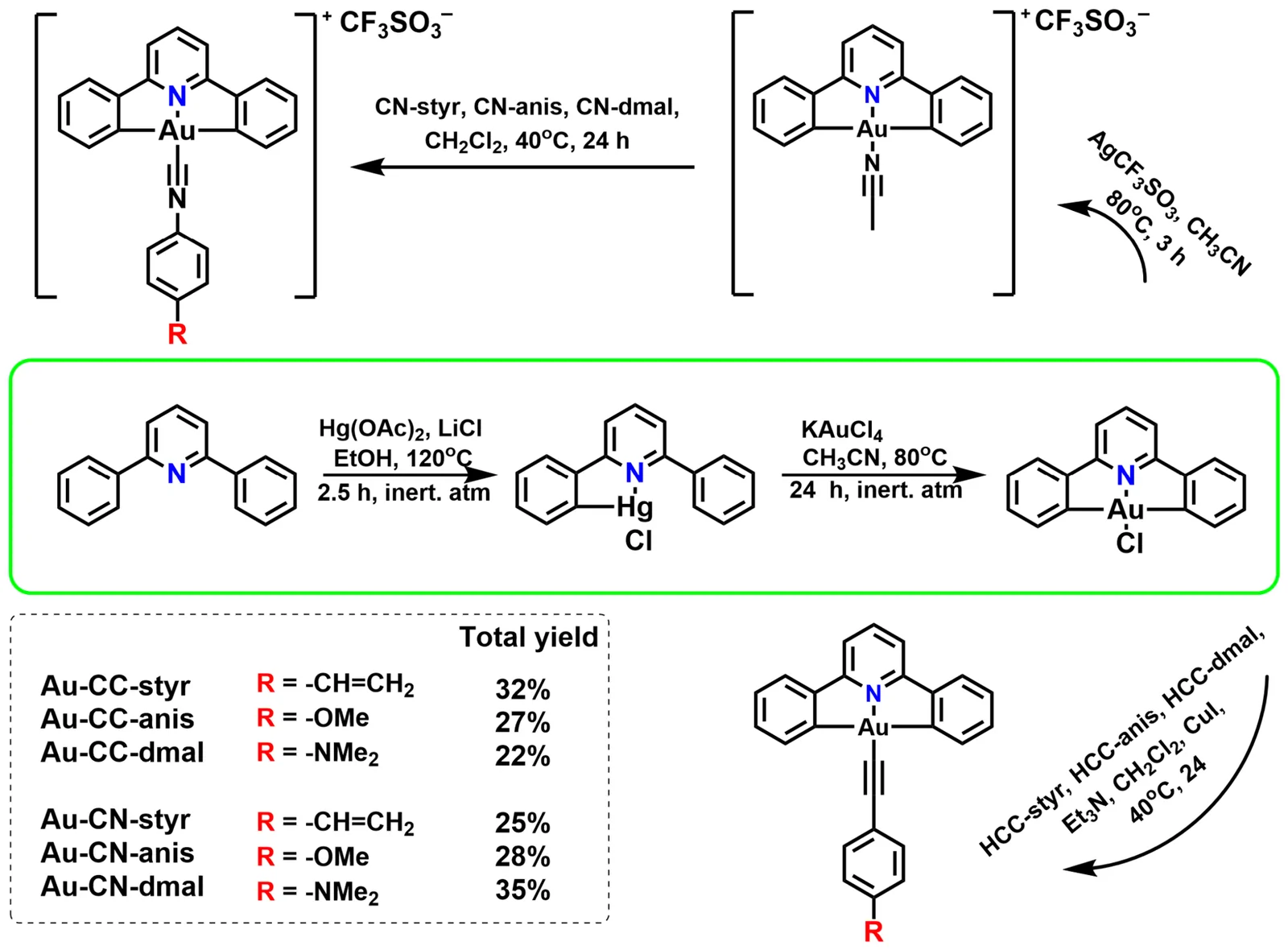
General synthetic procedure.

**Figure 1:**
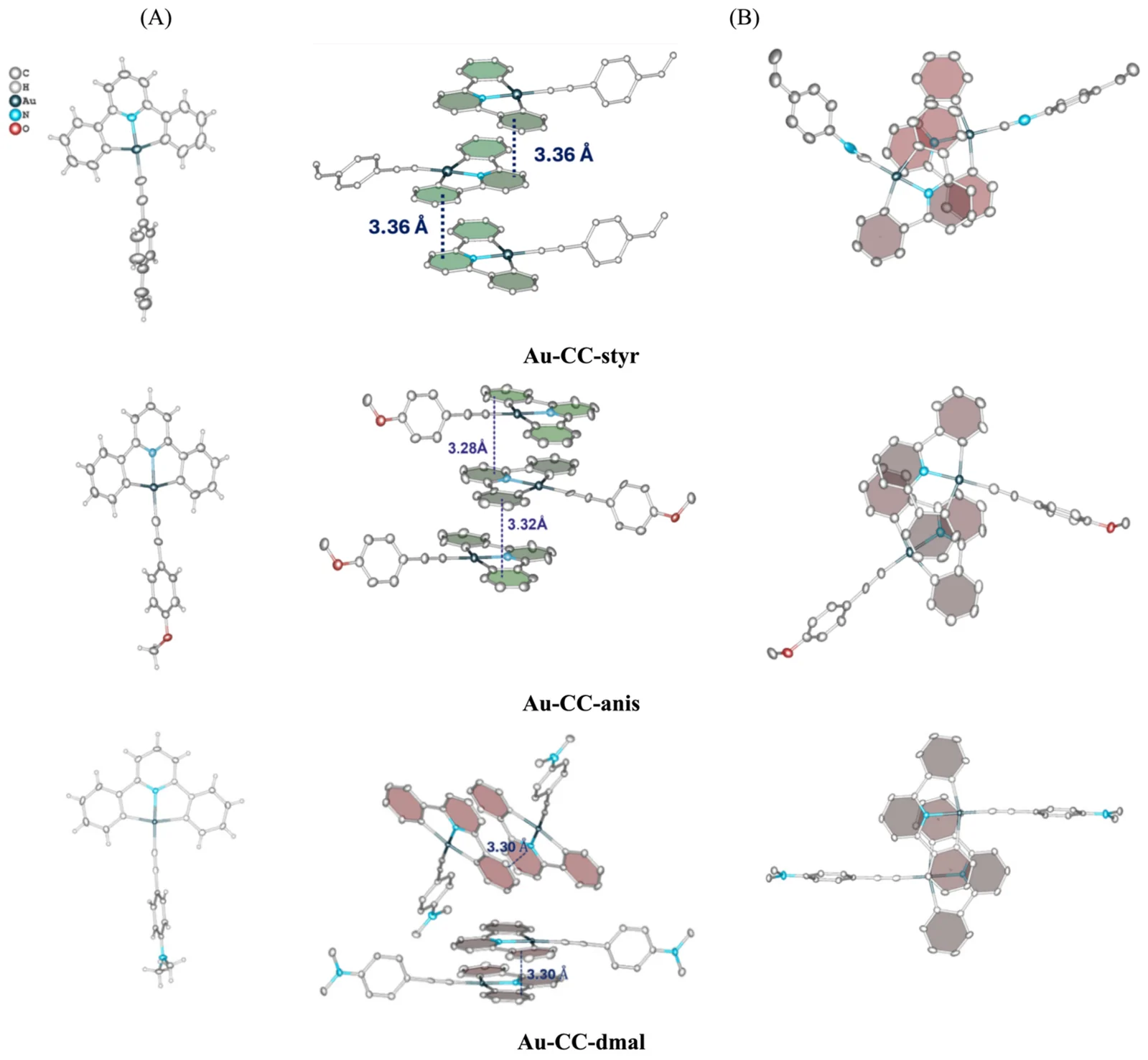
(**A**) Molecular views and (**B**) packing patterns of the alkynyl complexes with displacement ellipsoids shown at the 50% probability level. Counterions are omitted for clarity.

**Figure 2:**
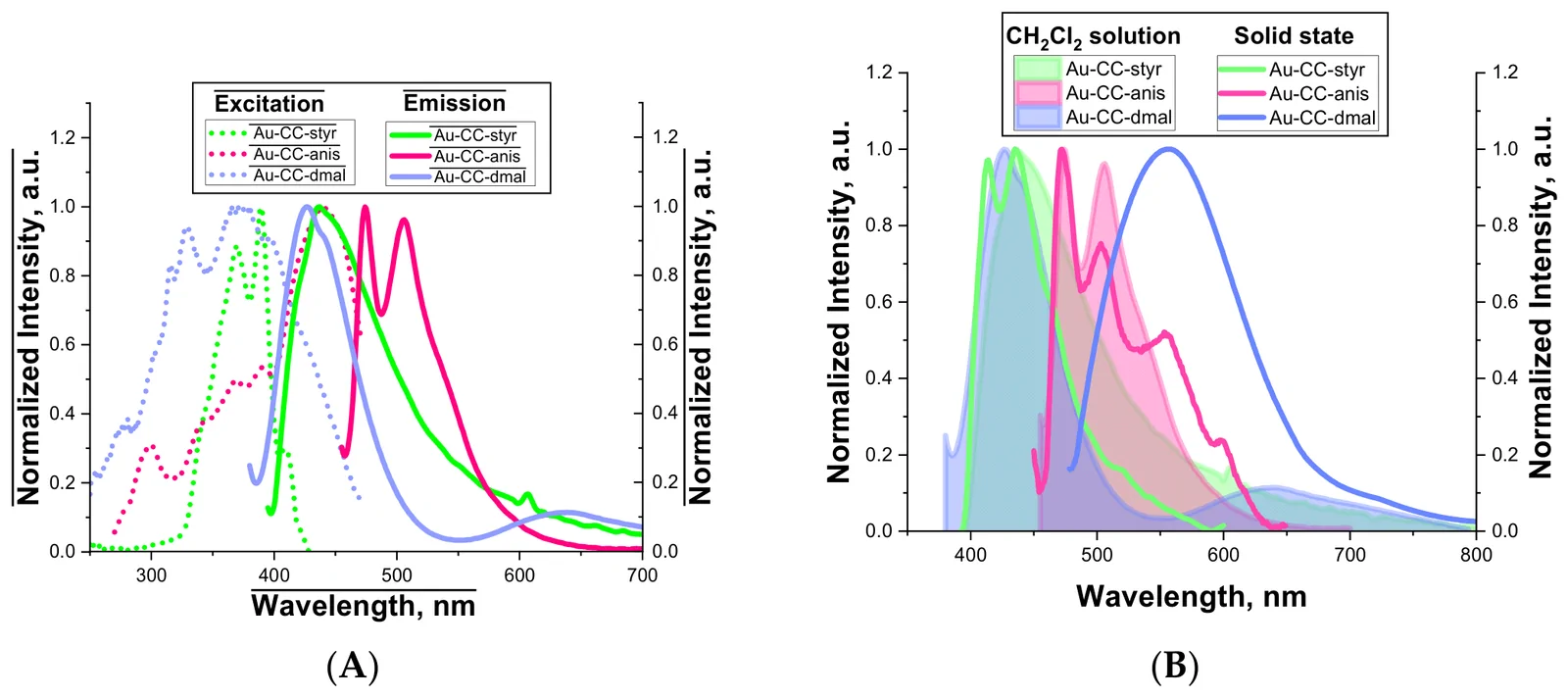
(**A**) Excitation and emission spectra of Au(III) alkynyl complexes in CH_2_Cl_2_ solution at concentration 10^−5^ mol/L. (**B**) Comparison of Au(III) alkynyl complexes emission spectra in solution and in solid state.

**Figure 3:**
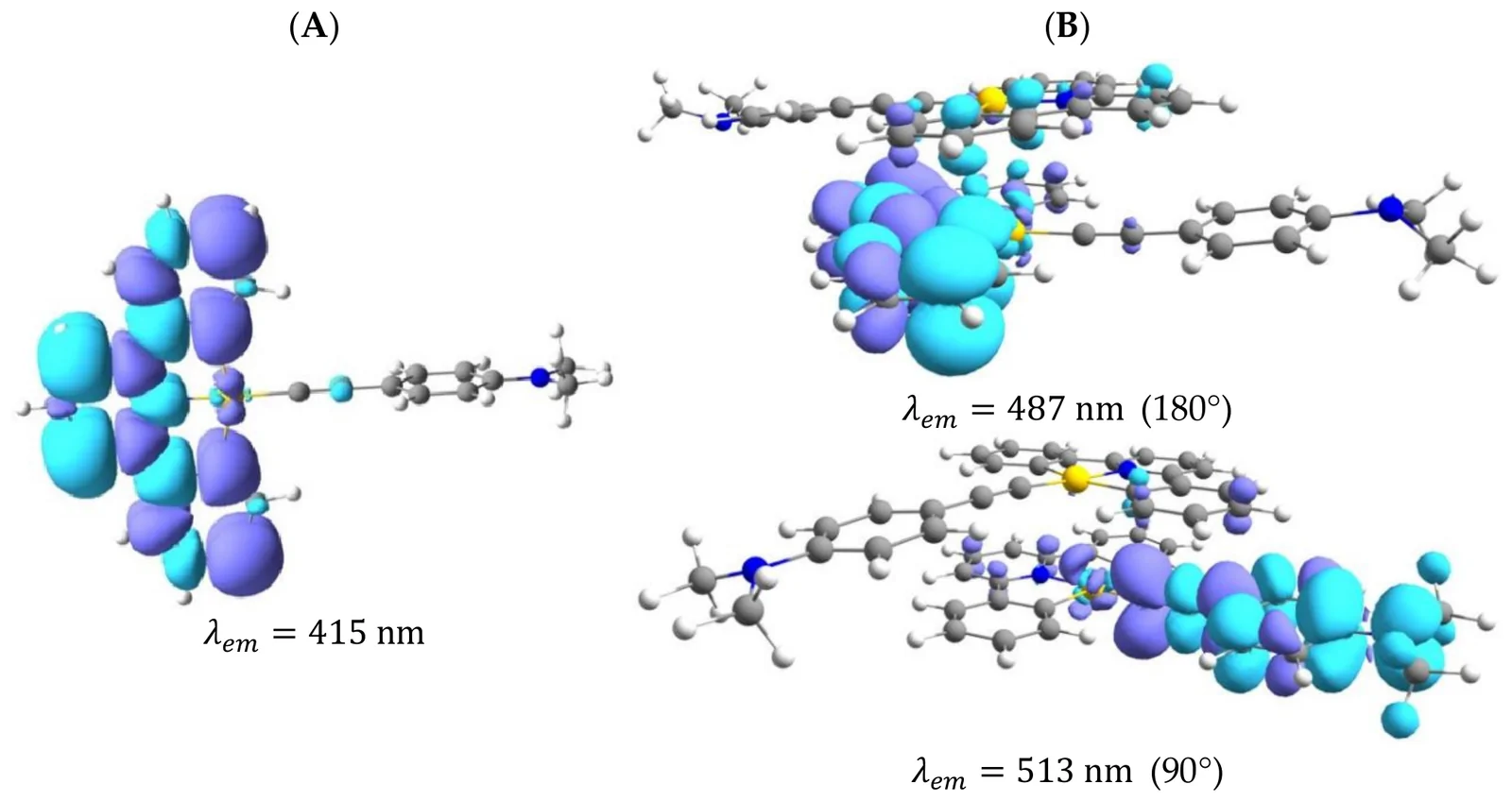
The decrease (violet) and increase (cyan) of electron density for the S0 → T1 transition calculated for **Au-CC-dmal** (**A**) monomer and (**B**) dimer in DCM and in gas-phase, respectively, at the M062X/(6-31g*, Au SDD) level of theory. The calculated emission maxima are given under the corresponding structures.

**Figure 4:**
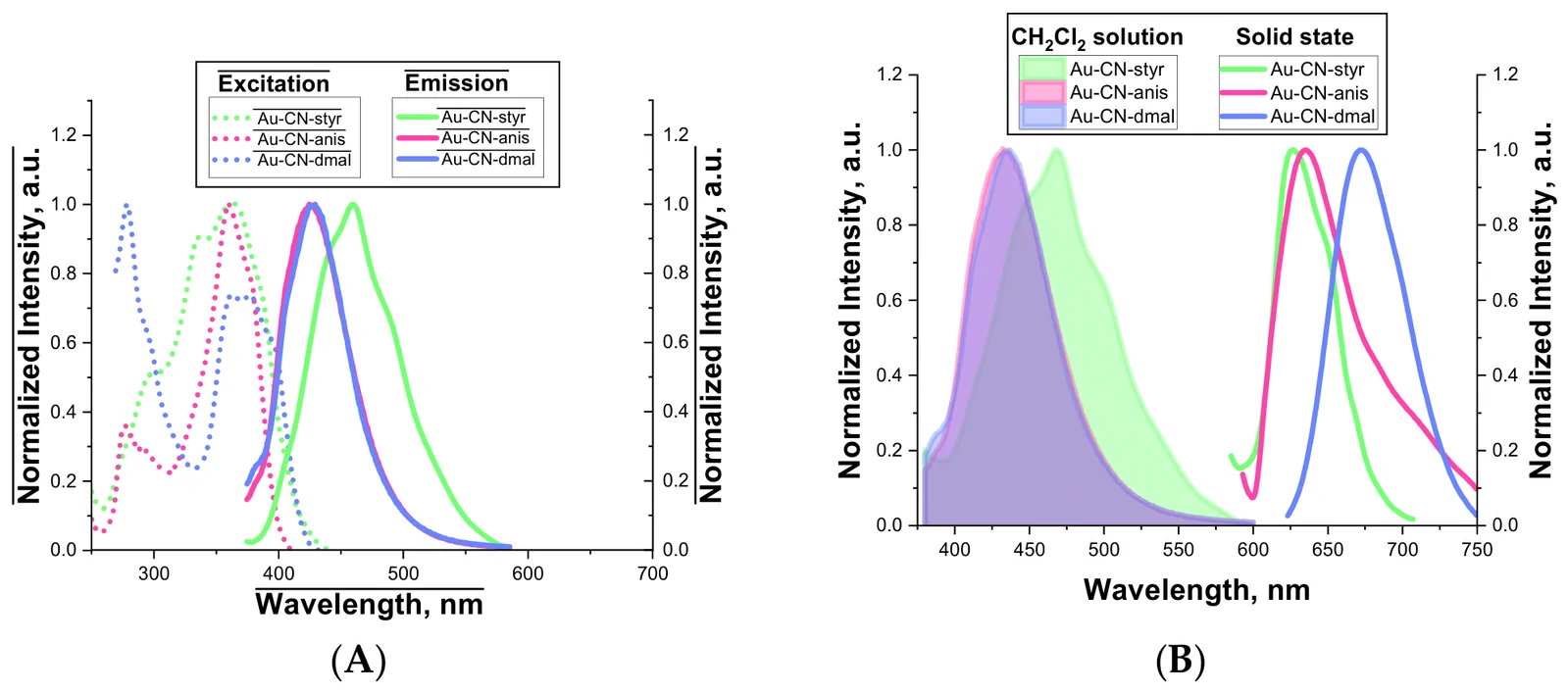
(**A**) Excitation and emission spectra of Au(III) isocyanide complexes in CH_2_Cl_2_ solution at concentration 10^−5^ mol/L. (**B**) Comparison of Au(III) isocyanide complexes emission spectra in solution and in solid state.

**Figure 5:**
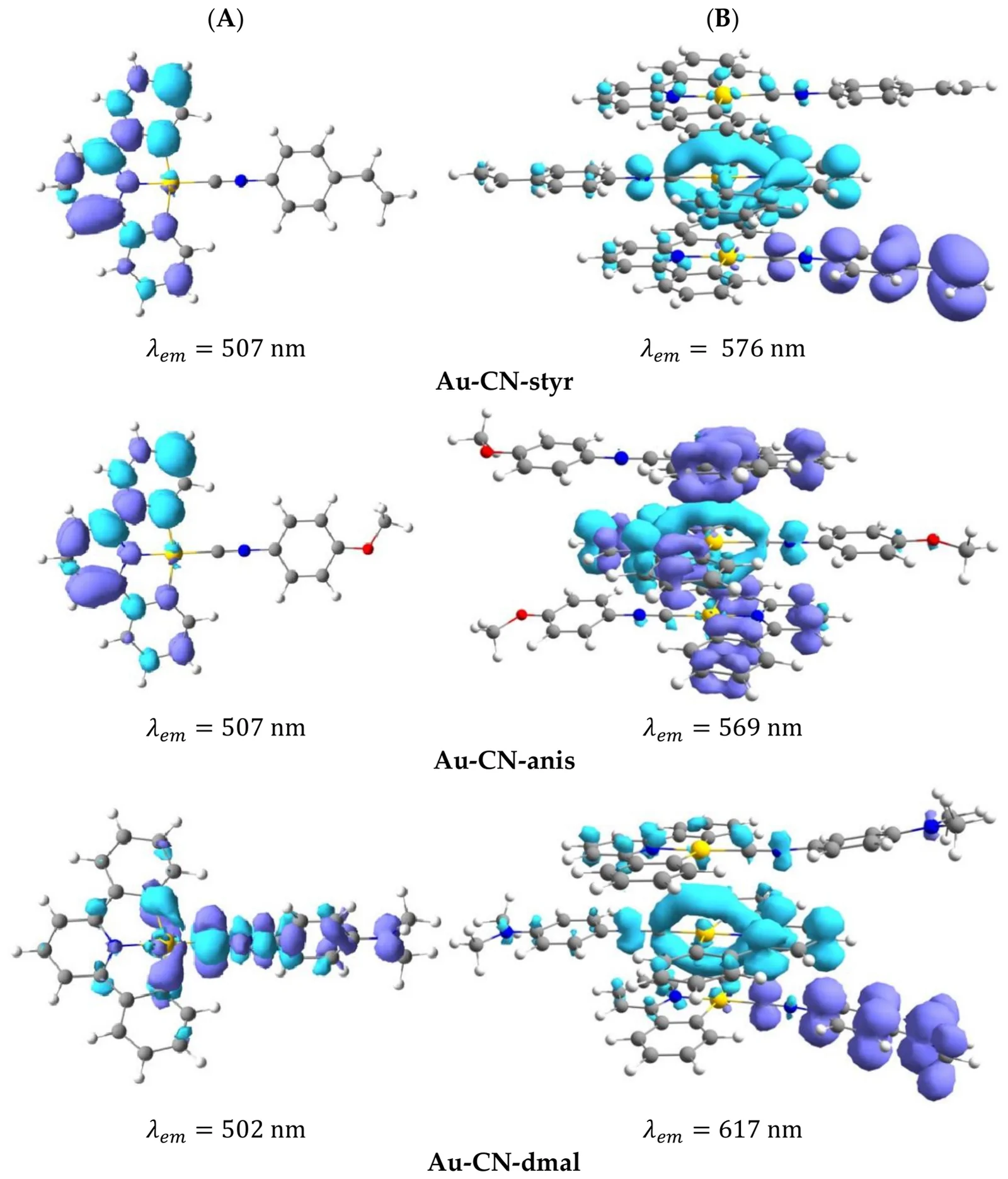
The decrease (violet) and increase (cyan) of electron density for the S0 → T1 transition calculated for (**A**) monomer and (**B**) dimer models of **Au-CN-styr**, **Au-CN-anis**, **Au-CN-dmal** complexes in the gas phase at the MN12SX/(6-31g*, Au SDD) level of theory. The calculated emission maxima are given under the corresponding structures.

**Figure 6:**
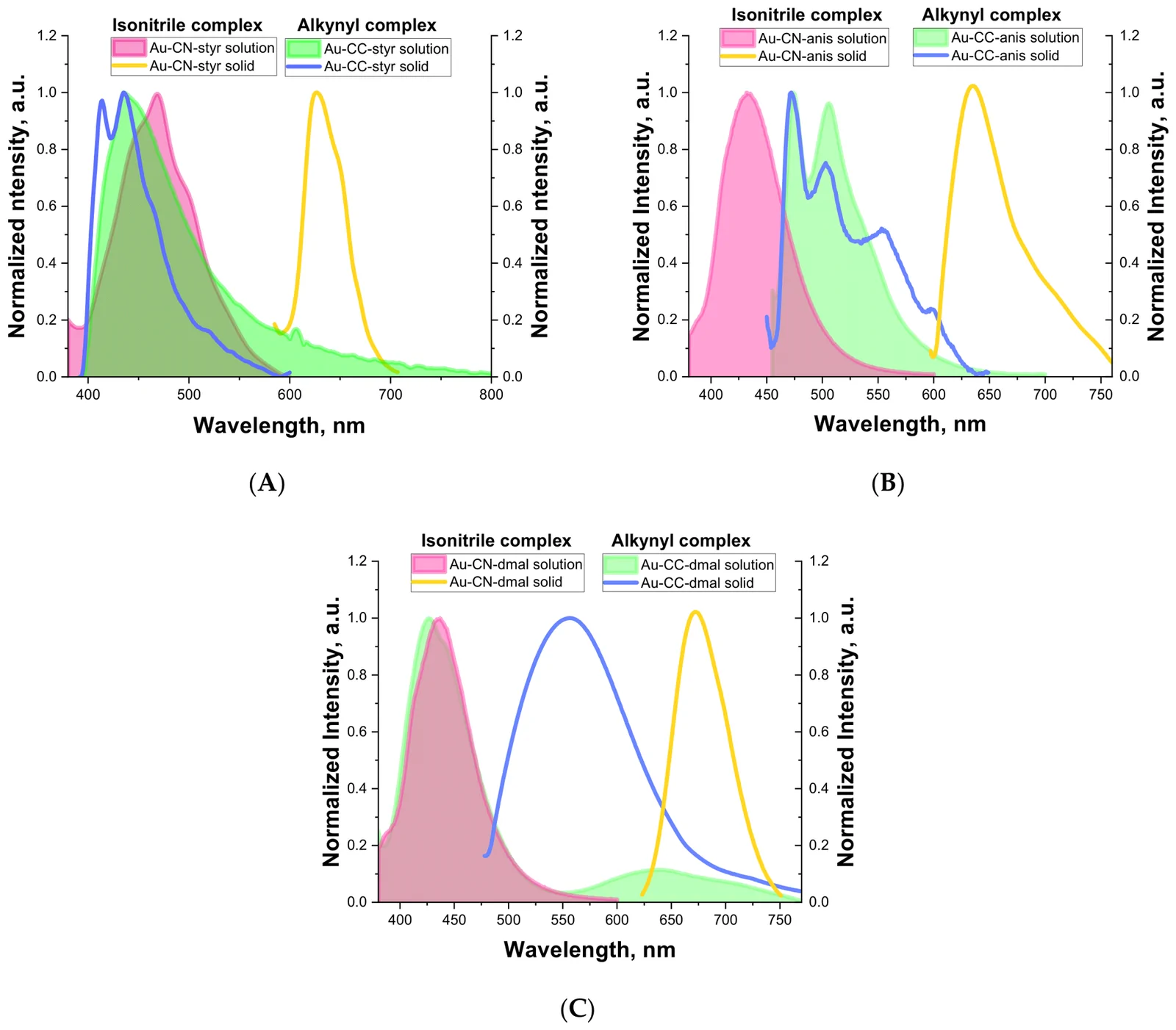
Comparison of emission spectra of **Au-CC-styr** vs. **Au-CN-styr** (**A**), Au-CC-anis vs. **Au-CN-anis** (**B**) and **Au-CC-dmal** vs. **Au-CN-dmal** (**C**) in CH_2_Cl_2_ solution (10^−5^ mol/L) and in the solid state.

**Table 1:** Photophysical properties of Au(III) complexes in CH_2_Cl_2_ solution at concentration 10^−5^ mol/L and solid phase.

		λ_abs_, nm	λ_exc_^[b]^, nm	λ_em_^[a]^, nm	Φ_aer/deg_, %	τ_deg_^[b,c]^, ns
**Au-CN-styr**	**DCM**	263, 375, 394	297sh, 340sh, 368	450sh, 468, 500sh	<0.1	22
	**Solid phase**	-	370	626	<0.1	149
**Au-CN-anis**	**DCM**	280, 315sh, 402, 423	279, 291sh, 363	433	0.4/0.9	33 ^[c]^
	**Solid phase**	-	368, 396	635	<0.1	69 ^[c]^
**Au-CN-dmal**	**DCM**	267sh, 285, 319, 398	265, 275, 363, 386sh	437	0.2/0.3	44 ^[c]^
	**Solid phase**	-	319, 366	672	<0.1	231
**Au-CC-styr**	**DCM**	262, 283, 311, 324sh, 381, 400	370, 390, 409	435	<0.1	^[d]^
	**Solid phase**	-	367	414, 435	<0.1	125
**Au-CC-anis**	**DCM**	281sh, 312, 322, 363, 379, 401	305, 347sh, 374, 392, 416	474, 506	0.1/0.2	398 ^[c]^
	**Solid phase**	-	282sh, 337, 361	472, 503, 554, 599	0.12	66
**Au-CC-dmal**	**DCM**	263sh, 285, 311sh, 364, 382, 400	359sh, 365, 379, 398, 417sh	426, 640	0.13/0.15	482
	**Solid phase**	-	278sh, 365, 405 sh	555, broad	<0.1	93 ^[c]^

^[a]^ λ_exc_ = 365 nm. ^[b]^ Excitation spectra and lifetimes were measured at the emission bands’ maxima. ^[c]^ The emission decay curves were fitted using a biexponential function. The relative contributions of the individual components are summarized in Table S3. ^[d]^ Emission is too weak to acquire the decay curve.

## Data Availability

The data supporting the findings of this study are available in the article and its Supplementary Materials. Crystallographic data for **Au-CC-styr**, **Au-CC-anis**, and **Au-CC-dmal** have been deposited to the Cambridge Crystallographic Data Centre (CCDC) under deposition numbers 2578199, 2578200, and 2578201, respectively, and are available free of charge from the CCDC database. Additional experimental and computational data are available from the corresponding author upon reasonable request.

## Supplementary Materials

The following supporting information can be downloaded at: https://www.mdpi.com/article/10.3390/molecules31193414/s1.
