# Supplementary material for: Synthesis and Photophysical Study of [(C^N^C)Au(L)]n+ Pincer Complexes, L = Alkynyl (n = 0), Isocyanide (n = +1): Comparative Analysis of Their Aggregation Behavior
Source: Molecules. 2026 Sep 25;31(19):3414. doi: 10.3390/molecules31193414 (PMC13665826; doi:10.3390/molecules31193414)

checkCIF/PLATON report

Structure factors have been supplied for datablock(s) au-cc-anis

THIS REPORT IS FOR GUIDANCE ONLY. IF USED AS PART OF A REVIEW PROCEDURE FOR PUBLICATION, IT SHOULD NOT REPLACE THE EXPERTISE OF AN EXPERIENCED CRYSTALLOGRAPHIC REFEREE.

No syntax errors found. CIF dictionary Interpreting this report

Datablock: au-cc-anis

Bond precision: C-C = 0.0137 A Wavelength=1.54184

Cell:	a=8.9570(3)	b=12.5989(4)	c=19.0018(4)
	alpha=77.267(2)	beta=78.172(2)	gamma=78.402(3)
Temperature:	100 K		

	Calculated	Reported
Volume	2019.71(11)	2019.71(11)
Space group	P -1	P -1
Hall group	-P 1	-P 1
Moiety formula	C26 H18 Au N O [+ solvent]	C26 H18 Au N O, 0.25[CH2CL2], 0.25[H2O]
Sum formula	C26 H18 Au N O [+ solvent]	C26.25 H19 Au Cl0.50 N O1.25
Mr	557.38	583.11
Dx,g cm-3	1.833	1.918
Z	4	4
Mu (mm-1)	13.809	14.447
F000	1072.0	1124.0
F000’	1056.68	
h,k,lmax	10,15,22	10,15,22
Nref	7281	7249
Tmin,Tmax	0.525,0.749	0.527,1.000
Tmin’	0.225	

Correction method= # Reported T Limits: Tmin=0.527 Tmax=1.000
AbsCorr = MULTI-SCAN

Data completeness= 0.996 Theta(max)= 67.496

R(reflections)= 0.0539(5936) wR2(reflections)= 0.1513(7249)

S = 1.113 Npar= 525

The following ALERTS were generated. Each ALERT has the format
test-name_ALERT_alert-type_alert-level.
Click on the hyperlinks for more details of the test.

Alert level B

PLAT971_ALERT_2_B Check Calcd Resid. Dens. 0.84Ang From Au01 . 3.12 eA-3

Author Response: A Larger than expected residual density maximum is due to the large weight of the gold atom.

PLAT971_ALERT_2_B Check Calcd Resid. Dens. 0.95Ang From Au02 . 3.09 eA-3

Author Response: A Larger than expected residual density maximum is due to the large weight of the gold atom.

PLAT971_ALERT_2_B Check Calcd Resid. Dens. 0.94Ang From Au01 . 3.05 eA-3

Author Response: A Larger than expected residual density maximum is due to the large weight of the gold atom.

PLAT971_ALERT_2_B Check Calcd Resid. Dens. 0.99Ang From Au01 . 3.01 eA-3

Author Response: A Larger than expected residual density maximum is due to the large weight of the gold atom.

PLAT971_ALERT_2_B Check Calcd Resid. Dens. 0.96Ang From Au02 . 2.94 eA-3

Author Response: A Larger than expected residual density maximum is due to the large weight of the gold atom.

PLAT971_ALERT_2_B Check Calcd Resid. Dens. 1.02Ang From Au02 . 2.77 eA-3

Author Response: A Larger than expected residual density maximum is due to the large weight of the gold atom.

```
PLAT971_ALERT_2_B Check Calcd Resid. Dens.  0.87Ang From Au01    .       2.51 eA-3
```

Author Response: A Larger than expected residual density maximum is due to the large weight of the gold atom.

```
PLAT972_ALERT_2_B Check Calcd Resid. Dens.  0.94Ang From Au02    .      -2.85 eA-3
```

Author Response: Larger than expected residual density minimum outside metal atom locations is due to possible errors during the experiment.

```
PLAT972_ALERT_2_B Check Calcd Resid. Dens.  1.02Ang From Au01    .      -2.52 eA-3
```

Author Response: Larger than expected residual density minimum outside metal atom locations is due to possible errors during the experiment.

```
PLAT973_ALERT_2_B Check Calcd Positive Resid. Density on      Au02       1.88 eA-3
```

Author Response: A Larger than expected residual density maximum is due to the large weight of the gold atom.

```
PLAT973_ALERT_2_B Check Calcd Positive Resid. Density on      Au01       1.77 eA-3
```

Author Response: A Larger than expected residual density maximum is due to the large weight of the gold atom.

Alert level C

```
PLAT342_ALERT_3_C Low Bond Precision on  C-C Bonds ...............    0.01375 Ang.
PLAT411_ALERT_2_C Short Inter H...H Contact  H01I      ..H01I     .       2.10 Ang.
                                               2-x,2-y,1-z  =      2_776 Check
PLAT413_ALERT_2_C Short Inter XH3 .. XHn     H015      ..H01C     .       2.06 Ang.
                                               2-x,2-y,2-z  =      2_777 Check
PLAT906_ALERT_3_C Large K Value in the Analysis of Variance ......      4.453 Check
PLAT911_ALERT_3_C Missing FCF Refl Between Thmin & STh/L=    0.599         32 Report
                1  1  0,  -4  9  0,   4 -8  1,   1  0  1,  -1 -3  2,  -1 -1  2,
                2  1  2,   0 -2  3,   0  0  3,   1  0  3,   2  0  3,   2  3  3,
                0 -2  4,   2  1  4,   0 -2  6,   6 13 10,  -8  1 11,  -7  3 12,
               -7  0 13,  -7  1 13,  -6  1 14,  -6  2 14,  -6  0 15,  -6  1 15,
               -5 -2 16,  -5  3 16,  -4  7 16,  -5  0 17,  -4  2 18,   2  4 18,
                  (    2 More NOT listed: see .ckf listing file)
PLAT934_ALERT_3_C Number of (Iobs-Icalc)/Sigma(W) > 10 Outliers ..          1 Check
               -5  4 15,
PLAT971_ALERT_2_C Check Calcd Resid. Dens.  0.80Ang From Au02    .       2.49 eA-3
```

Author Response: A Larger than expected residual density maximum is due to the large weight of the gold atom.

```
PLAT971_ALERT_2_C Check Calcd Resid. Dens.  0.96Ang From Au01    .       1.74 eA-3
```

Author Response: A Larger than expected residual density maximum is due to the large weight of the gold atom.

```
PLAT971_ALERT_2_C Check Calcd Resid. Dens.  1.04Ang From Au02    .       1.58 eA-3
```

Author Response: A Larger than expected residual density maximum is due to the large weight of the gold atom.

```
PLAT972_ALERT_2_C Check Calcd Resid. Dens.  0.86Ang From Au01    .      -2.23 eA-3
```

Author Response: Larger than expected residual density minimum outside metal atom locations is due to possible errors during the experiment.

```
PLAT972_ALERT_2_C Check Calcd Resid. Dens.  0.89Ang From Au02    .      -2.21 eA-3
```

Author Response: Larger than expected residual density minimum outside metal atom locations is due to possible errors during the experiment.

```
PLAT972_ALERT_2_C Check Calcd Resid. Dens.  0.73Ang From Au01    .      -1.55 eA-3
```

Author Response: Larger than expected residual density minimum outside metal atom locations is due to possible errors during the experiment.

```
PLAT977_ALERT_2_C Check Negative Difference Density on H00W      .      -0.31 eA-3
```

Alert level G

```
FORMU01_ALERT_1_G  There is a discrepancy between the atom counts in the
            _chemical_formula_sum and _chemical_formula_moiety. This is
            usually due to the moiety formula being in the wrong format.
            Atom count from _chemical_formula_sum:   C26.25 H19 Au1 Cl0.5 N1 O1.25
            Atom count from _chemical_formula_moiety:C26.25 H18.5 Au1 Cl0.5 N1 O1
FORMU01_ALERT_2_G  There is a discrepancy between the atom counts in the
            _chemical_formula_sum and the formula from the _atom_site* data.
            Atom count from _chemical_formula_sum:C26.25 H19 Au1 Cl0.5 N1 O1.25
            Atom count from the _atom_site data:  C26 H18 Au1 N1 O1
CELLZ01_ALERT_1_G Difference between formula and atom_site contents detected.
CELLZ01_ALERT_1_G ALERT: Large difference may be due to a
            symmetry error - see SYMMG tests
           From the CIF: _cell_formula_units_Z    4
           From the CIF: _chemical_formula_sum  C26.25 H19 Au Cl0.50 N O1.25
           TEST: Compare cell contents of formula and atom_site data

           atom    Z*formula  cif sites diff
           C        105.00    104.00    1.00
           H         76.00     72.00    4.00
           Au         4.00      4.00    0.00
```

```
          Cl         2.00      0.00    2.00
          N          4.00      4.00    0.00
          O          5.00      4.00    1.00
PLAT041_ALERT_1_G Calc. and Reported SumFormula    Strings  Differ     Please Check
             Calc: C26 H18 Au N O
             Rep.: C26.25 H19 Au Cl0.50 N O1.25
PLAT042_ALERT_1_G Calc. and Reported MoietyFormula Strings  Differ     Please Check
             Calc: C26 H18 Au N O
             Rep.: C26 H18 Au N O, 0.25[CH2CL2], 0.25[H2O]
PLAT051_ALERT_1_G Mu(calc) and Mu(cif) Ratio Differs from 1.0 by .       4.42 %
PLAT083_ALERT_2_G SHELXL Second Parameter in WGHT  Unusually Large       8.58 Why ?
PLAT371_ALERT_2_G Long   C(sp2)-C(sp1) Bond  C00T     - C013     .       1.43 Ang.
PLAT371_ALERT_2_G Long   C(sp2)-C(sp1) Bond  C00N     - C01F     .       1.43 Ang.
PLAT605_ALERT_4_G Largest Solvent Accessible VOID in the Structure         99 A**3
PLAT720_ALERT_4_G Number of Unusual/Non-Standard Labels ..........         94 Note
             Au01    Au02    O004    O005    N006    N007    C008    C009
             C00A    H00A    C00B    C00C    C00D    H00D    C00E    H00E
             C00F    C00G    H00G    C00H    C00I    C00J    C00K    C00L
             C00M    H00M    C00N    C00O    C00P    C00Q    H00Q    C00R
             H00R    C00S    H00S    C00T    C00U    H00U    C00V    H00V
             C00W    H00W    C00X    H00X    C00Y    H00Y    C00Z    H00Z
             C010    C011    H011    C012    H012    C013    C014    H01E
             H01F    H01N    C015    H015    C016    C017    H017    C018
             H018    C019    H019    C01A    C01B    H01B    C01C    C01D
             H01O    C01E    H01P    C01F    C01G    H01G    C01H    H01H
             C01I    H01I    C01J    H01J    C01K    H01K    C01L    H01L
             C01M    H01M    C01N    H01A    H01C    H01D
PLAT868_ALERT_4_G ALERTS Due to the Use of _smtbx_masks Suppressed          ! Info
PLAT909_ALERT_3_G Percentage of I>2sig(I) Data at Theta(Max) Still        72% Note
PLAT933_ALERT_2_G Number of HKL-OMIT Records in Embedded .res File         34 Note
              -7 -2 14,  -7 -1 14,  -7  0 13,  -7  3 12,  -6 -1 16,  -6  0 15,
              -6  0 16,  -6  1 14,  -6  1 15,  -6  1 16,  -6  2 14,  -6  2 16,
              -6  6 14,  -5  0 17,  -5  2 18,  -5  3 16,  -4  0 19,  -4  2 18,
              -4  6 18,  -4  7 16,  -1 -3  2,  -1 -1  2,   0 -2  3,   0 -2  4,
               0 -2  6,   0  0  3,   1  0  1,   1  0  3,   1  1  0,   2  0  3,
               2  1  2,   2  1  4,   2  3  3,   7 -9  4,
PLAT941_ALERT_3_G Average HKL Measurement Multiplicity ..........        4.2 Low
PLAT967_ALERT_5_G Note: Two-Theta Cutoff Value in Embedded .res ..      135.0 Degree
PLAT969_ALERT_5_G The 'Henn et al.' R-Factor-gap value ..........      3.061 Note
             Predicted wR2: Based on SigI**2  4.94 or SHELX Weight 13.59 Note
PLAT978_ALERT_2_G Number C-C Bonds with Positive Residual Density.          0 Info
```

```
   0 ALERT level A = Most likely a serious problem - resolve or explain
  11 ALERT level B = A potentially serious problem, consider carefully
  13 ALERT level C = Check. Ensure it is not caused by an omission or oversight
  19 ALERT level G = General information/check it is not something unexpected

   6 ALERT type 1 CIF construction/syntax error, inconsistent or missing data
  26 ALERT type 2 Indicator that the structure model may be wrong or deficient
   6 ALERT type 3 Indicator that the structure quality may be low
   3 ALERT type 4 Improvement, methodology, query or suggestion
   2 ALERT type 5 Informative message, check
```

It is advisable to attempt to resolve as many as possible of the alerts in all categories. Often the minor alerts point to easily fixed oversights, errors and omissions in your CIF or refinement strategy, so attention to these fine details can be worthwhile. It is up to the individual to critically assess their own results and, if necessary, seek expert advice.

PLATON version of 20/06/2026; check.def file version of 19/06/2026

Datablock au-cc-anis - ellipsoid plot

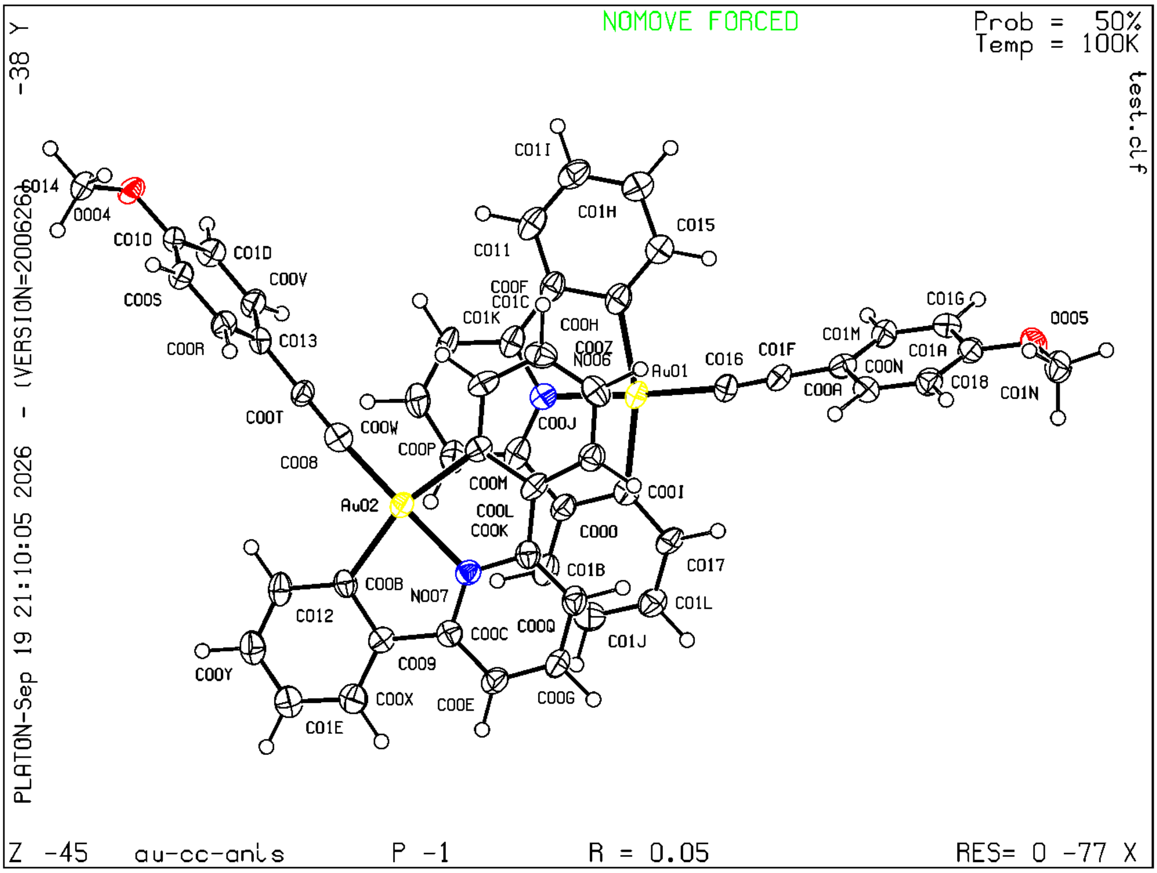

Supplement: Supplementary file 1 [file molecules-31-03414-s001.zip › checkcif_Au-CC-anis.pdf]
