# Supplementary material for: Synthesis and Photophysical Study of [(C^N^C)Au(L)]n+ Pincer Complexes, L = Alkynyl (n = 0), Isocyanide (n = +1): Comparative Analysis of Their Aggregation Behavior
Source: Molecules. 2026 Sep 25;31(19):3414. doi: 10.3390/molecules31193414 (PMC13665826; doi:10.3390/molecules31193414)

checkCIF/PLATON report

Structure factors have been supplied for datablock(s) au-cc-styr

THIS REPORT IS FOR GUIDANCE ONLY. IF USED AS PART OF A REVIEW PROCEDURE FOR PUBLICATION, IT SHOULD NOT REPLACE THE EXPERTISE OF AN EXPERIENCED CRYSTALLOGRAPHIC REFEREE.

No syntax errors found. CIF dictionary Interpreting this report

Datablock: au-cc-styr

Bond precision: C-C = 0.0101 A Wavelength=1.54184

Cell:	a=9.4656(1)	b=16.8984(2)	c=21.2564(2)
	alpha=95.879(1)	beta=99.239(1)	gamma=90.000(1)
Temperature:	100 K		

	Calculated	Reported
Volume	3337.81(6)	3337.81(6)
Space group	P -1	P -1
Hall group	-P 1	-P 1
Moiety formula	3(C27 H18 Au N), 2(C3 H6 O)	3(C27 H18 Au N), 2(C3 H6 O)
Sum formula	C87 H66 Au3 N3 O2	C87 H66 Au3 N3 O2
Mr	1776.34	1776.32
Dx,g cm-3	1.768	1.767
Z	2	2
Mu (mm-1)	12.565	12.565
F000	1724.0	1724.0
F000'	1701.19	
h,k,lmax	12,21,27	12,21,26
Nref	14625	13946
Tmin,Tmax	0.465,0.686	0.451,1.000
Tmin'	0.170	

Correction method= # Reported T Limits: Tmin=0.451 Tmax=1.000
AbsCorr = MULTI-SCAN

```
Data completeness= 0.954          Theta(max)= 80.409

R(reflections)= 0.0451( 12636)                       wR2(reflections)=
                                                     0.1261( 13946)
S = 1.063                  Npar= 860
```

The following ALERTS were generated. Each ALERT has the format
test-name_ALERT_alert-type_alert-level.
Click on the hyperlinks for more details of the test.

Alert level B

```
SYMMS02_ALERT_1_B  No unit-cell angle should be equal to 90 for a triclinic
            cell
            Cell       9.4656   16.8984   21.2564
            Angles    95.8790   99.2390   90.0000
```

Author Response: The triclinic space group was retained because the gamma angle of 90.0 degrees arises from accidental metric pseudosymmetry, while no additional crystallographic symmetry was detected.

```
PLAT971_ALERT_2_B Check Calcd Resid. Dens.  0.78Ang From C008    .       2.53 eA-3
```

Author Response: The residual electron-density peak located 0.78 Ang from C008 is attributed to minor unresolved disorder, and its modelling as an additional atomic site did not produce a chemically meaningful or stable refinement.

Alert level C

```
PLAT213_ALERT_2_C Atom C008           has ADP max/min Ratio .....        3.6 prolat
PLAT244_ALERT_4_C Low  MinorResAtom  Ueq as Compared to Neighbours       C02E Check
PLAT244_ALERT_4_C Low  MinorResAtom  Ueq as Compared to Neighbours       C02H Check
PLAT342_ALERT_3_C Low Bond Precision on  C-C Bonds ..............    0.01007 Ang.
PLAT411_ALERT_2_C Short Inter H...H Contact  H022     ..H02F     .       2.14 Ang.
                                              -1+x,y,-1+z  =      1_454 Check
PLAT911_ALERT_3_C Missing FCF Refl Between Thmin & STh/L=    0.600         19 Report
               -4  6  0,   5-18  1,   4 -6  1,   3 -4  1,   5-18  2,  -4 -6  2,
                0 -3  2,  -1 -2  2,   0  2  2,   0  3  2,  -6 16  2,   5-18  3,
               -2 -2  3,  -3  1  3,  -1 -2  4,  -2  5  5,   0 -6  6,  -1 -1  7,
               -5 15 11,
PLAT971_ALERT_2_C Check Calcd Resid. Dens.  1.02Ang From Au02    .       1.86 eA-3
```

Author Response: The residual electron-density peak located 0.78 Ang from C008 is attributed to minor unresolved disorder, and its modelling as an additional atomic site did not produce a chemically meaningful or stable refinement.

PLAT971_ALERT_2_C Check Calcd Resid. Dens. 0.79Ang From Au01 . 1.79 eA-3

Author Response: The residual electron-density peak located 0.78 Ang from C008 is attributed to minor unresolved disorder, and its modelling as an additional atomic site did not produce a chemically meaningful or stable refinement.

PLAT971_ALERT_2_C Check Calcd Resid. Dens. 0.82Ang From Au02 . 1.75 eA-3

Author Response: The residual electron-density peak located 0.78 Ang from C008 is attributed to minor unresolved disorder, and its modelling as an additional atomic site did not produce a chemically meaningful or stable refinement.

PLAT971_ALERT_2_C Check Calcd Resid. Dens. 1.07Ang From Au03 . 1.71 eA-3

Author Response: The residual electron-density peak located 0.78 Ang from C008 is attributed to minor unresolved disorder, and its modelling as an additional atomic site did not produce a chemically meaningful or stable refinement.

PLAT971_ALERT_2_C Check Calcd Resid. Dens. 0.88Ang From Au01 . 1.69 eA-3

Author Response: The residual electron-density peak located 0.78 Ang from C008 is attributed to minor unresolved disorder, and its modelling as an additional atomic site did not produce a chemically meaningful or stable refinement.

PLAT971_ALERT_2_C Check Calcd Resid. Dens. 0.84Ang From Au01 . 1.65 eA-3

Author Response: The residual electron-density peak located 0.78 Ang from C008 is attributed to minor unresolved disorder, and its modelling as an additional atomic site did not produce a chemically meaningful or stable refinement.

PLAT971_ALERT_2_C Check Calcd Resid. Dens. 1.02Ang From Au02 . 1.64 eA-3

Author Response: The residual electron-density peak located 0.78 Ang from C008 is attributed to minor unresolved disorder, and its modelling as an additional atomic site did not produce a chemically meaningful or stable refinement.

PLAT971_ALERT_2_C Check Calcd Resid. Dens. 0.98Ang From Au03 . 1.58 eA-3

Author Response: The residual electron-density peak located 0.78 Ang from C008 is attributed to minor unresolved disorder, and its modelling as an additional atomic site did not produce a chemically meaningful or stable refinement.

```
PLAT972_ALERT_2_C Check Calcd Resid. Dens.  0.76Ang From Au02    .      -2.13 eA-3
PLAT972_ALERT_2_C Check Calcd Resid. Dens.  0.81Ang From Au02    .      -2.06 eA-3
PLAT972_ALERT_2_C Check Calcd Resid. Dens.  0.84Ang From Au01    .      -2.05 eA-3
PLAT972_ALERT_2_C Check Calcd Resid. Dens.  0.87Ang From Au01    .      -1.90 eA-3
PLAT972_ALERT_2_C Check Calcd Resid. Dens.  0.77Ang From Au02    .      -1.84 eA-3
PLAT972_ALERT_2_C Check Calcd Resid. Dens.  0.81Ang From Au03    .      -1.82 eA-3
PLAT972_ALERT_2_C Check Calcd Resid. Dens.  0.82Ang From Au02    .      -1.81 eA-3
PLAT972_ALERT_2_C Check Calcd Resid. Dens.  0.85Ang From Au03    .      -1.75 eA-3
PLAT972_ALERT_2_C Check Calcd Resid. Dens.  0.75Ang From Au01    .      -1.73 eA-3
PLAT972_ALERT_2_C Check Calcd Resid. Dens.  0.77Ang From Au03    .      -1.62 eA-3
PLAT972_ALERT_2_C Check Calcd Resid. Dens.  0.81Ang From Au01    .      -1.61 eA-3
PLAT972_ALERT_2_C Check Calcd Resid. Dens.  0.82Ang From Au01    .      -1.52 eA-3
PLAT973_ALERT_2_C Check Calcd Positive Resid. Density on      Au01       1.21 eA-3
PLAT973_ALERT_2_C Check Calcd Positive Resid. Density on      Au03       1.07 eA-3
```

Alert level G

```
PLAT002_ALERT_2_G Number of Distance or Angle Restraints on AtSite          2 Note
PLAT083_ALERT_2_G SHELXL Second Parameter in WGHT  Unusually Large       7.66 Why ?
PLAT154_ALERT_1_G The s.u.'s on the Cell Angles are Equal ..(Note)      0.001 Degree
PLAT172_ALERT_4_G The CIF-Embedded .res File Contains DFIX Records          1 Report
PLAT230_ALERT_2_G Hirshfeld Test Diff for    C4       --C020     .       11.2 s.u.
PLAT232_ALERT_2_G Hirshfeld Test Diff (M-X)  Au02     --C008     .       20.0 s.u.
PLAT371_ALERT_2_G Long   C(sp2)-C(sp1) Bond  C00U     - C01T     .       1.43 Ang.
PLAT371_ALERT_2_G Long   C(sp2)-C(sp1) Bond  C00D     - C00Q     .       1.45 Ang.
PLAT371_ALERT_2_G Long   C(sp2)-C(sp1) Bond  C4       - C020     .       1.43 Ang.
PLAT720_ALERT_4_G Number of Unusual/Non-Standard Labels ..........        160 Note
              Au01    Au02    Au03    N004    N005    N006    C007    C008
              C009    C00A    C00B    C00C    C00D    C00E    C00F    C00G
              C00H    C00I    C00J    C00K    C00L    H00L    C00M    C00N
              C00O    H00O    C00P    C00Q    C00R    H00R    C00S    C00T
              H00T    C00U    C00V    H00V    C00W    H00W    C00X    C00Y
              H00Y    C00Z    H00Z    C010    H010    C011    C012    C013
              C014    H014    C015    H015    C016    H016    C017    H017
              C018    H018    C019    H019    C01A    H01A    C01B    H01B
              C01C    C01D    H01D    C01E    H01E    C01F    H01F    C01G
              H01G    O01H    C01I    H01I    C01J    H01J    C01K    H01K
              C01L    H01L    C01M    H01M    C01N    H01N    C01O    H01O
              C01P    H01P    C01Q    C01R    H01R    C01S    H01S    C01T
              C01U    H01U    C01V    H01V    C01W    H01W    C01X    H01X
              C01Y    H01Y    C01Z    H01Z    C020    C021    H021    C022
              H022    C023    H023    O024    C025    H025    C026    H026
              C027    H027    C028    C029    H029    C02A    H02C    C02B
              H02H    C02C    H02D    C02D    H02A    H02B    C02E    C02F
              H02I    H02J    C02G    H02G    C02H    C02I    H02Q    H02R
              H02S    C02J    H02E    H02F    C02K    H02K    H02L    H02M
              C02L    H02T    H02U    H02V    C02M    H02N    H02O    H02P
PLAT802_ALERT_4_G CIF Input Record(s) with more than 80 Characters          1 Info
PLAT860_ALERT_3_G Number of Least-Squares Restraints .............          1 Note
PLAT912_ALERT_4_G Missing # of FCF Reflections Above STh/L=  0.600        617 Note
PLAT933_ALERT_2_G Number of HKL-OMIT Records in Embedded .res File         31 Note
              -12  0  6, -11  1 13, -11  3 12, -11  4 12, -11  5 11, -11  6 10,
              -11  6 11, -11  7  4, -11  8  0, -11  8  5, -11  8  6,  -5-11 23,
               -3  1  3,  -2 -2  3,  -2  5  5,  -1 -2  2,  -1 -2  4,  -1 -1  7,
                0 -6  6,   0 -3  2,   0  2  2,   0  3  2,   1-10 24,   3-19  9,
                3-19 10,   3-16 17,   3 -4  1,   4-19  7,   4  5 22,   5-18  7,
                5-18  8,
```

```
PLAT941_ALERT_3_G Average HKL Measurement Multiplicity ..........        3.8 Low
PLAT953_ALERT_1_G Reported (CIF) and Actual (FCF) Hmax Differ by .         1 Units
PLAT969_ALERT_5_G The 'Henn et al.' R-Factor-gap value ..........      3.121 Note
              Predicted wR2: Based on SigI**2  4.04 or SHELX Weight 11.87 Note
PLAT978_ALERT_2_G Number C-C Bonds with Positive Residual Density.         1 Info
```

```
   0 ALERT level A = Most likely a serious problem - resolve or explain
   2 ALERT level B = A potentially serious problem, consider carefully
  28 ALERT level C = Check. Ensure it is not caused by an omission or oversight
  18 ALERT level G = General information/check it is not something unexpected

   3 ALERT type 1 CIF construction/syntax error, inconsistent or missing data
  34 ALERT type 2 Indicator that the structure model may be wrong or deficient
   4 ALERT type 3 Indicator that the structure quality may be low
   6 ALERT type 4 Improvement, methodology, query or suggestion
   1 ALERT type 5 Informative message, check
```

It is advisable to attempt to resolve as many as possible of the alerts in all categories. Often the minor alerts point to easily fixed oversights, errors and omissions in your CIF or refinement strategy, so attention to these fine details can be worthwhile. It is up to the individual to critically assess their own results and, if necessary, seek expert advice.

PLATON version of 20/06/2026; check.def file version of 19/06/2026

Datablock au-cc-styr - ellipsoid plot

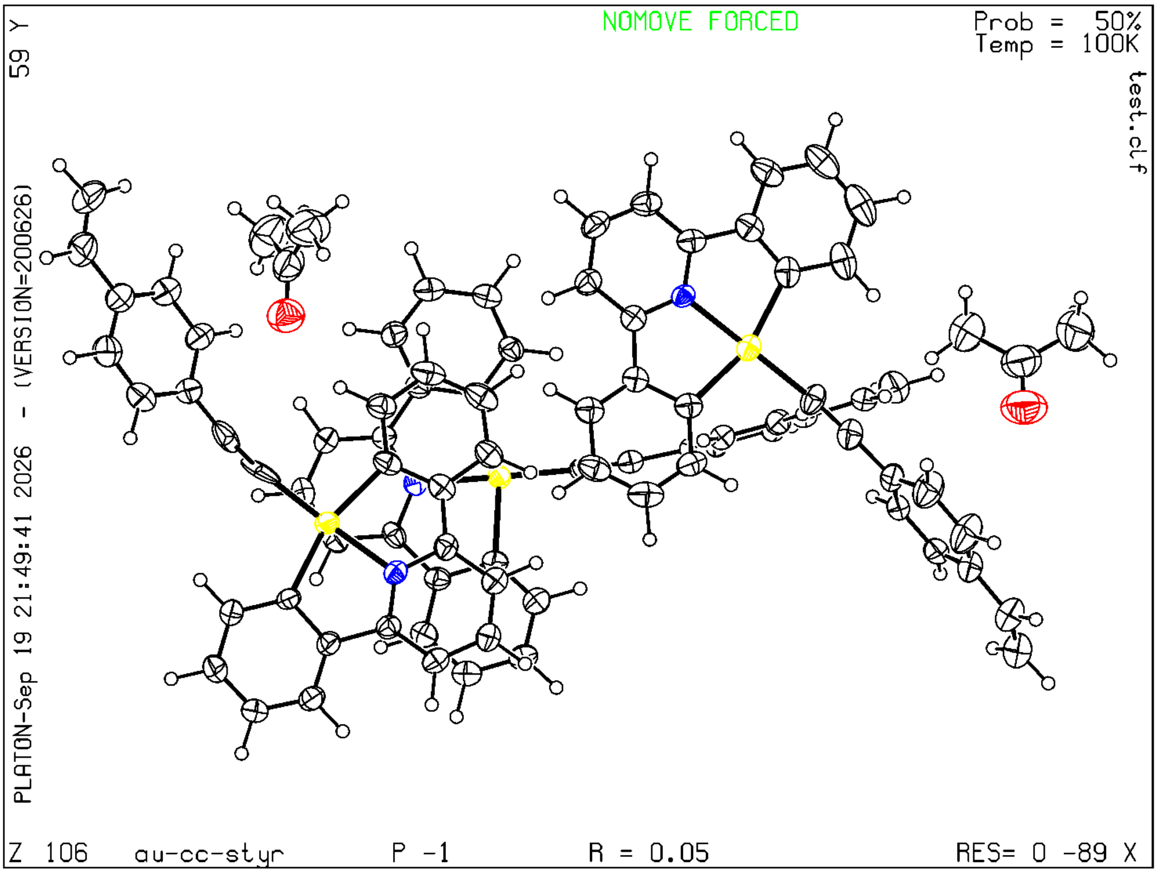

Supplement: Supplementary file 1 [file molecules-31-03414-s001.zip › checkcif_Au-CC-styr.pdf]
