# Supplementary material for: Synthesis and Photophysical Study of [(C^N^C)Au(L)]n+ Pincer Complexes, L = Alkynyl (n = 0), Isocyanide (n = +1): Comparative Analysis of Their Aggregation Behavior
Source: Molecules. 2026 Sep 25;31(19):3414. doi: 10.3390/molecules31193414 (PMC13665826; doi:10.3390/molecules31193414)
Supplement: Supplementary file 1 [file molecules-31-03414-s001.zip › Supplementary figures and tables.pdf]

Supporting Information
for the article

Synthesis and Photophysical Study of $[(C\text{^}N\text{^}C)Au(L)]^{n+}$ Pincer Complexes, L = Alkynyl (n = 0), Isocyanide (n = +1): Comparative Analysis of Their Aggregation Behavior

*Elizaveta V. Durova, Arina S. Stroeva, Kristina S. Kisel, Mikhail A. Kinzhalov, Viktor V. Sokolov, Georgiy A. Shonin, Dmitrii M. Nikolaev, Mikhail N. Ryazantsev, Sergey P. Tunik **

Institute of Chemistry, Saint-Petersburg State University, Universitetskii pr., 26, 198504 Saint-Petersburg, Russia

Content

S1. X-ray Structure Determination **.......... 2**

S2. HR-ESI$^+$, MALDI MS and NMR Spectra **.......... 4**

S3. Photophysical data **.......... 12**

S4. Calculations **.......... 15**

S1. X-ray Structure Determination

Table S1. Crystal data and structure refinement for **Au-CC-styr**, **Au-CC-anis**, **Au-CC-dmal**.

Identification code	**Au-CC-styr**	**Au-CC-anis**	**Au-CC-dmal**
Empirical formula	$C_{29}H_{22}AuNO_{0.67}$	$C_{52.5}H_{38}Au_2ClN_2O_{2.5}$	$C_{27}H_{21}AuN_2$
Formula weight	592.11	1191.26	570.42
Temperature/K	100(2)	100(2)	100(2)
Crystal system	triclinic	triclinic	monoclinic
Space group	P-1	P-1	$P2_1/c$
a/Å	9.46560(10)	8.9570(3)	15.30460(10)
b/Å	16.8984(2)	12.5989(4)	11.78000(10)
c/Å	21.2564(2)	19.0018(4)	11.67660(10)
α/°	95.8790(10)	77.267(2)	90
β/°	99.2390(10)	78.172(2)	103.8300(10)
γ/°	90.0000(10)	78.402(3)	90
Volume/$Å^3$	3337.81(6)	2019.71(11)	2044.12(3)
Z	2	4	4
$\rho_{calc}g/cm^3$	1.767	1.959	1.854
μ/mm^{-1}	12.565	14.481	13.637
F(000)	1724	1152	1104
Crystal size/mm^3	0.13 × 0.06 × 0.03	0.10 × 0.04 × 0.02	0.21 × 0.05 × 0.03
Radiation	Cu Kα (λ = 1.54184)		
2Θ range for data collection/°	4.234 to 160.818	7.292 to 142.338	5.948 to 141.17
Index ranges	$-12 \le h \le 11, -19 \le k \le 21, -26 \le l \le 26$	$-10 \le h \le 10, -15 \le k \le 15, -22 \le l \le 23$	$-18 \le h \le 18, -14 \le k \le 14, -13 \le l \le 14$
Reflections collected	52716	31545	22361
Independent reflections	13946 [R_{int} = 0.0538, R_{sigma} = 0.0429]	7668 [R_{int} = 0.0746, R_{sigma} = 0.0552]	3908 [R_{int} = 0.0387, R_{sigma} = 0.0230]
Data/restraints/parameters	13946/1/860	7668/0/532	3908/0/273
Goodness-of-fit on F^2	1.071	1.098	1.062
Final R indexes [I>=2σ (I)]	R_1 = 0.0451, wR_2 = 0.1216	R_1 = 0.0539, wR_2 = 0.1750	R_1 = 0.0223, wR_2 = 0.0542
Final R indexes [all data]	R_1 = 0.0491, wR_2 = 0.1254	R_1 = 0.0858, wR_2 = 0.1866	R_1 = 0.0229, wR_2 = 0.0551
Largest diff. peak/hole / e $Å^{-3}$	2.60/-2.13	9.12/-2.87	1.59/-1.20
CCDC	2578199	2578200	2578201

Table S2. Major structural parameters for **Au-CC-styr**, **Au-CC-anis**, **Au-CC-dmal**.

	**Au-CC-styr**	**Au-CC-anis**	**Au-CC-dmal**
**Bond length, Å**			
**Au-C_1**	2.068(6)	2.068(11)	2.067(5)
**Au-C_2**	2.078(5)	2.072(11)	2.063(4)
**Au-C_3**	1.979(5)	1.972(11)	1.988(5)
**Au-N**	2.008(5)	2.019(9)	2.005(4)
**Closest Au-Au intermolecular distance**	5.8733(3)	4.2758(6)	5.4045(3)
**Angles, °**			
**C_2-Au-C_3**	98.9(2)	102.0(4)	97.60(18)
**C_2-Au-C_1**	162.9(2)	162.2(5)	100.06(18)
**C_1-Au-N**	81.0(2)	81.1(4)	81.06(17)
**N-Au-C_3**	179.1(2)	176.1(4)	176.57(17)

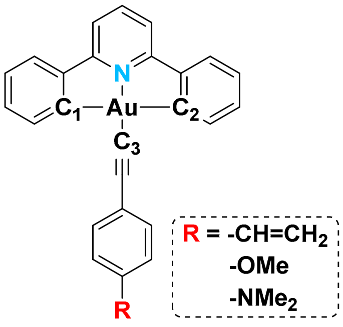

S2. HR-ESI$^+$, MALDI MS and NMR Spectra

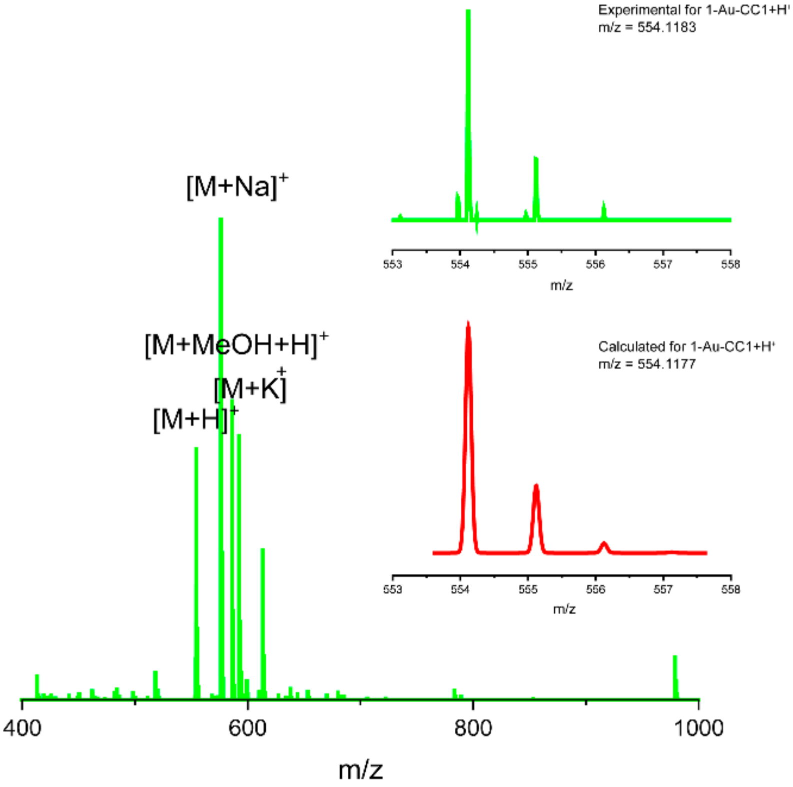

Figure S1. HR-ESI$^+$ mass-spectrum of **Au-CC-styr** with calculated isotopic pattern for the major signal of $[C_{27}H_{19}AuN]^+$ molecular ion.

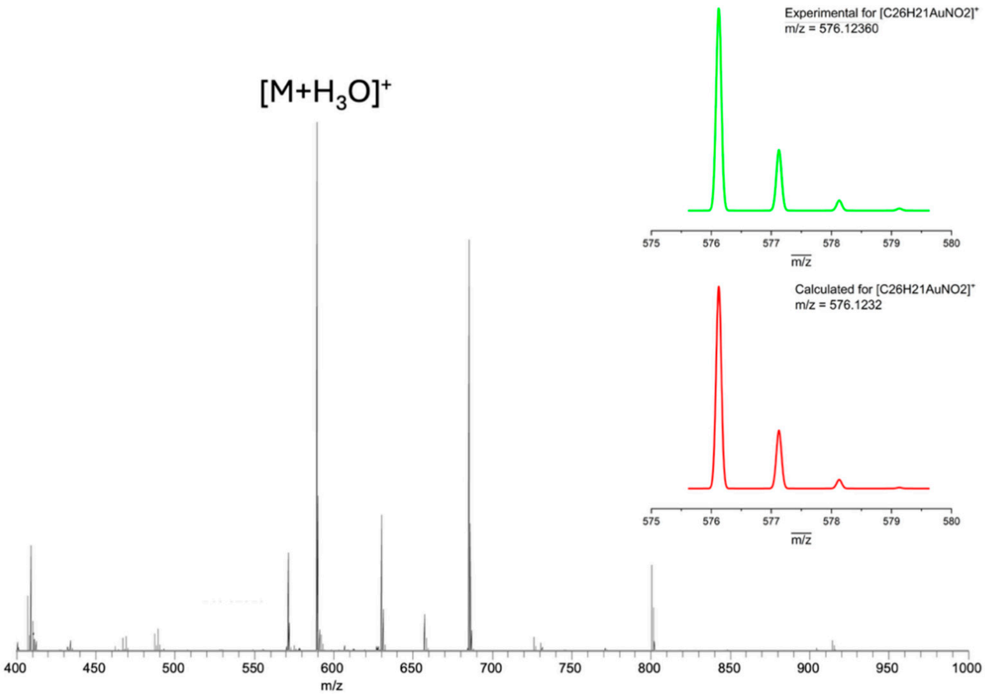

Figure S2. HR-ESI$^+$ mass-spectrum of **Au-CC-anis** with calculated isotopic pattern for the major signal of $[C_{26}H_{21}AuNO_2]^+$ adducts.

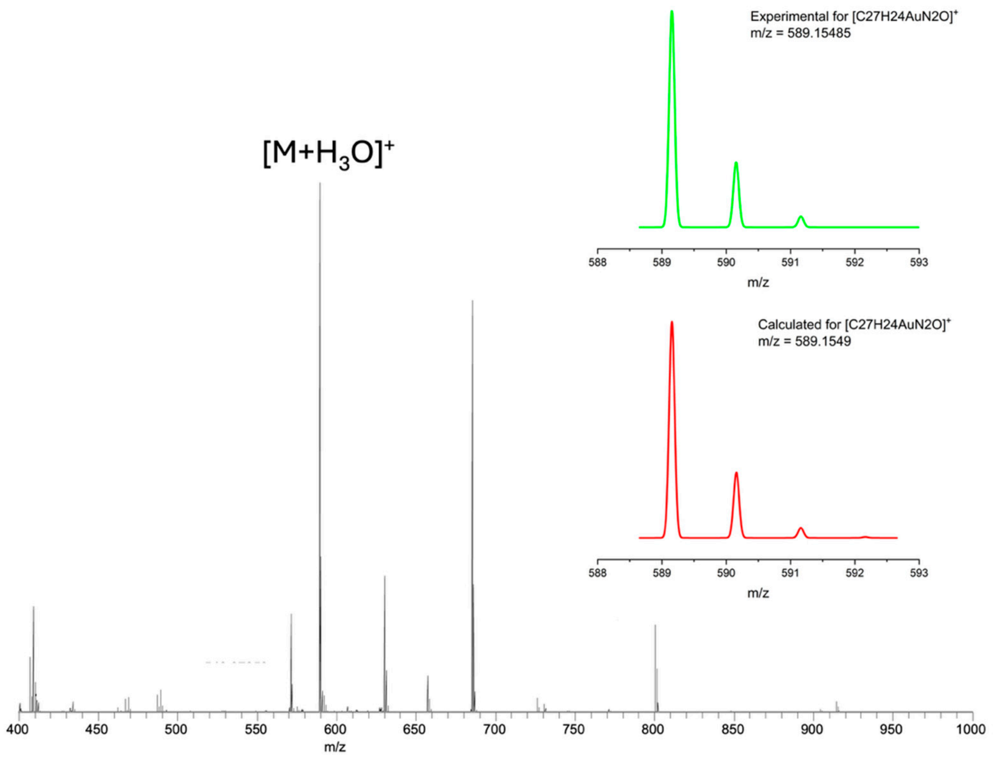

Figure S3. HR-ESI+ mass-spectrum of **Au-CC-dmal** with calculated isotopic pattern for the major signal of [C27H24AuN2O]+ molecular ion.

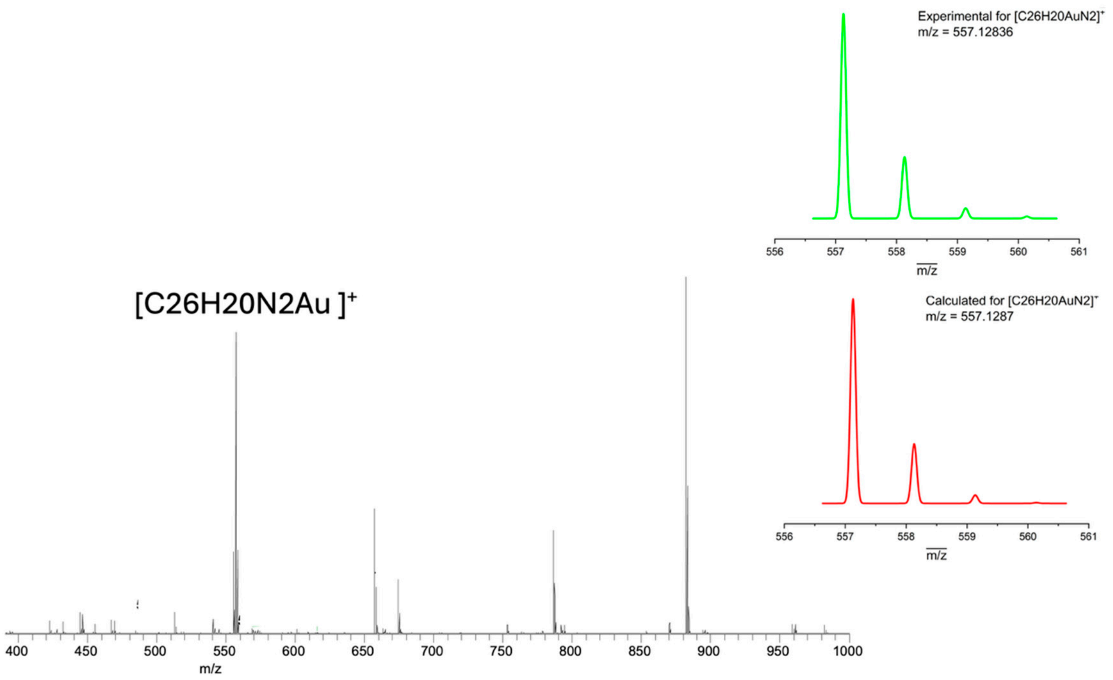

-

Figure S4. HR-ESI+ mass-spectrum of **Au-CN-styr** with calculated isotopic pattern for the major signal of [$C_{26}H_{20}N_2Au$]+ molecular ion.

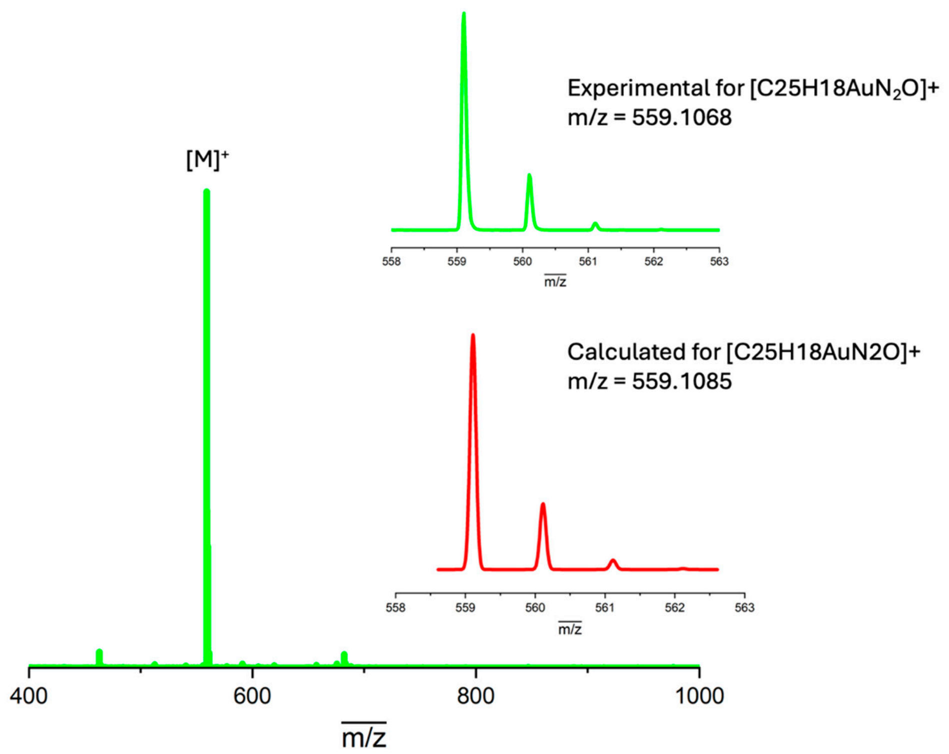

Figure S5. MALDI mass-spectrum of **Au-CN-anis** with calculated isotopic pattern for the major signal of $[C_{25}H_{18}AuN_2O]^+$ molecular ion.

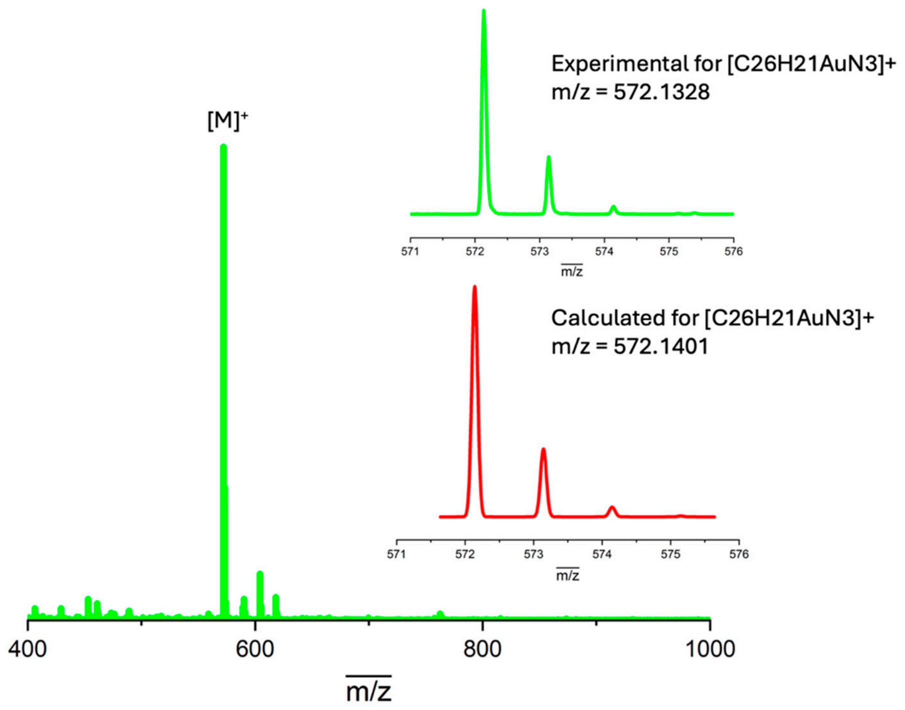

Figure S6. MALDI mass-spectrum of **Au-CN-dmal** with calculated isotopic pattern for the major signal of $[C_{26}H_{21}AuN_3]^+$ molecular ion.

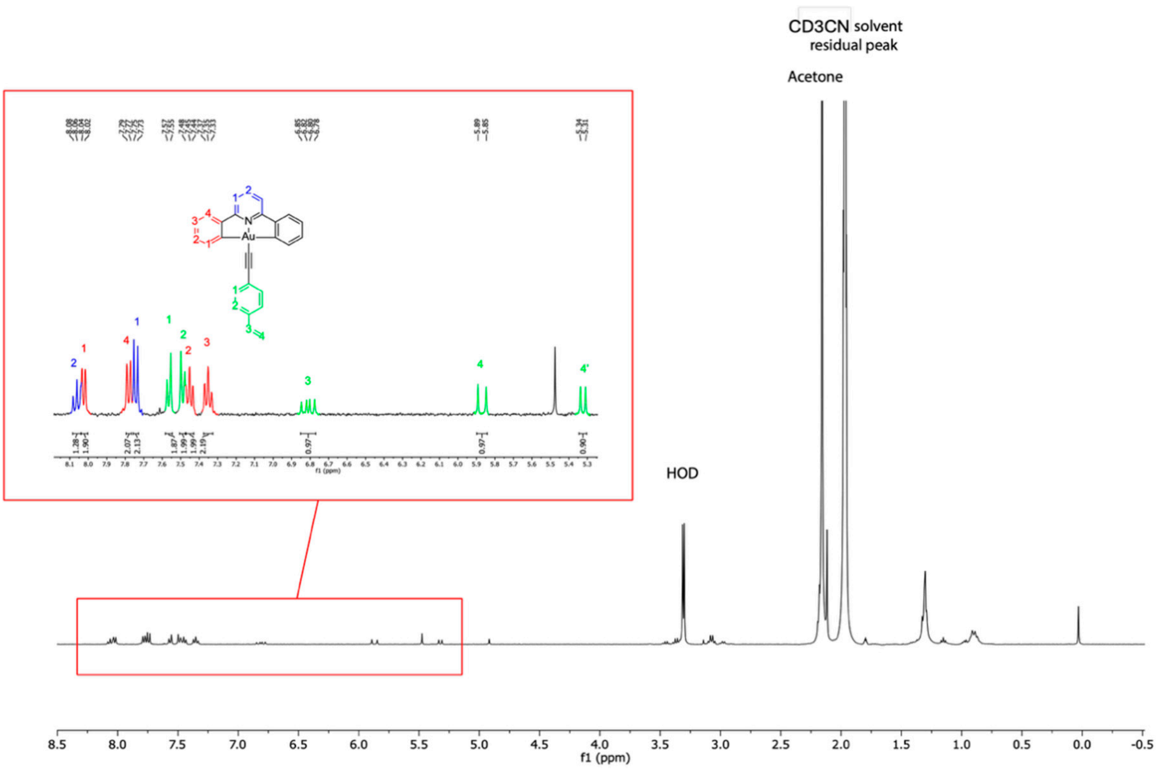

Figure S7. ^{1}H NMR spectrum of **Au-CC-styr**, 400 MHz, acetonitrile-d3, 25ºC.

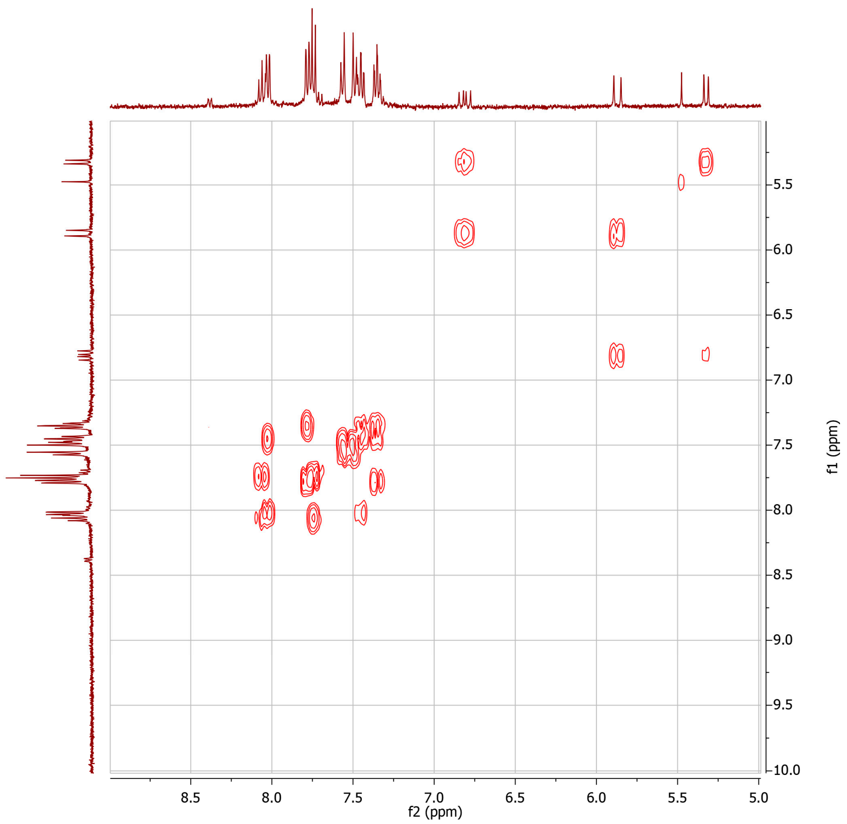

Figure S8. ^{1}H-^{1}H COSY NMR spectrum of **Au-CC-styr**, 400 MHz, acetonitrile-d3, 25ºC.

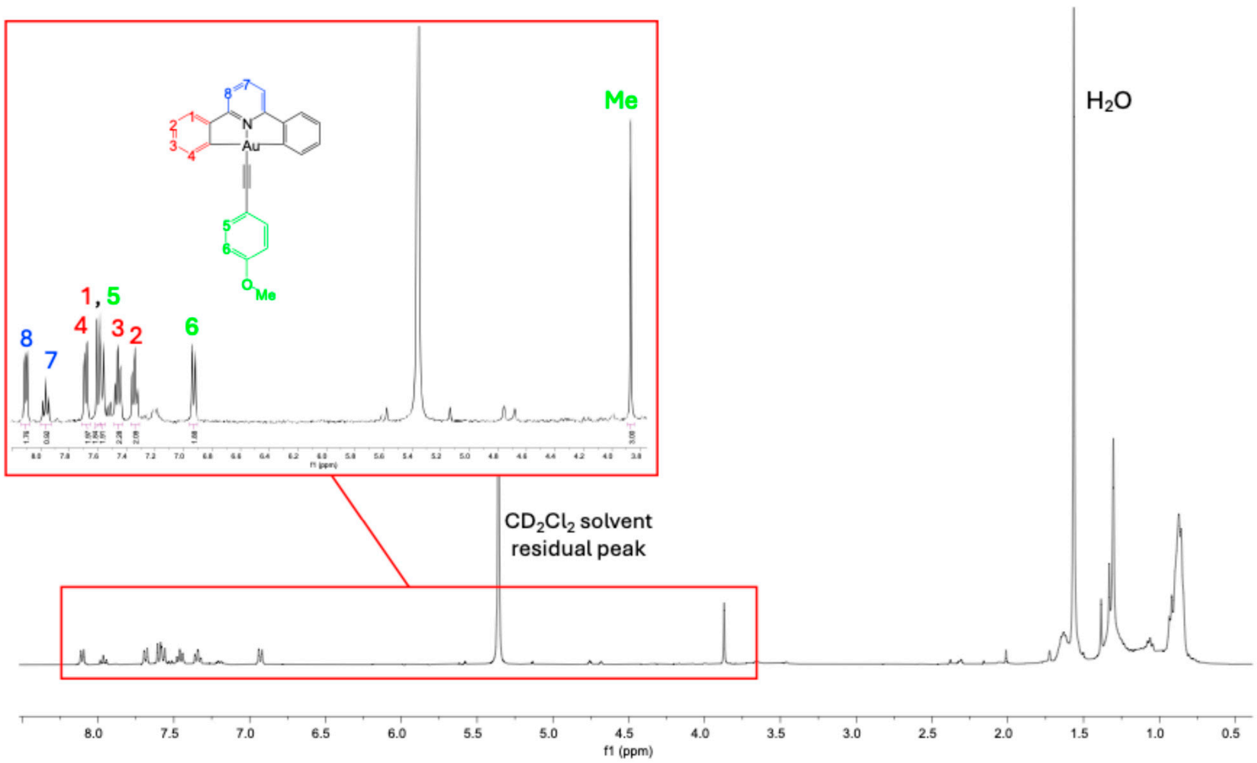

Figure S9. ^{1}H NMR spectrum of **Au-CC-anis**, 400 MHz, CD_2Cl_2, 25°C.

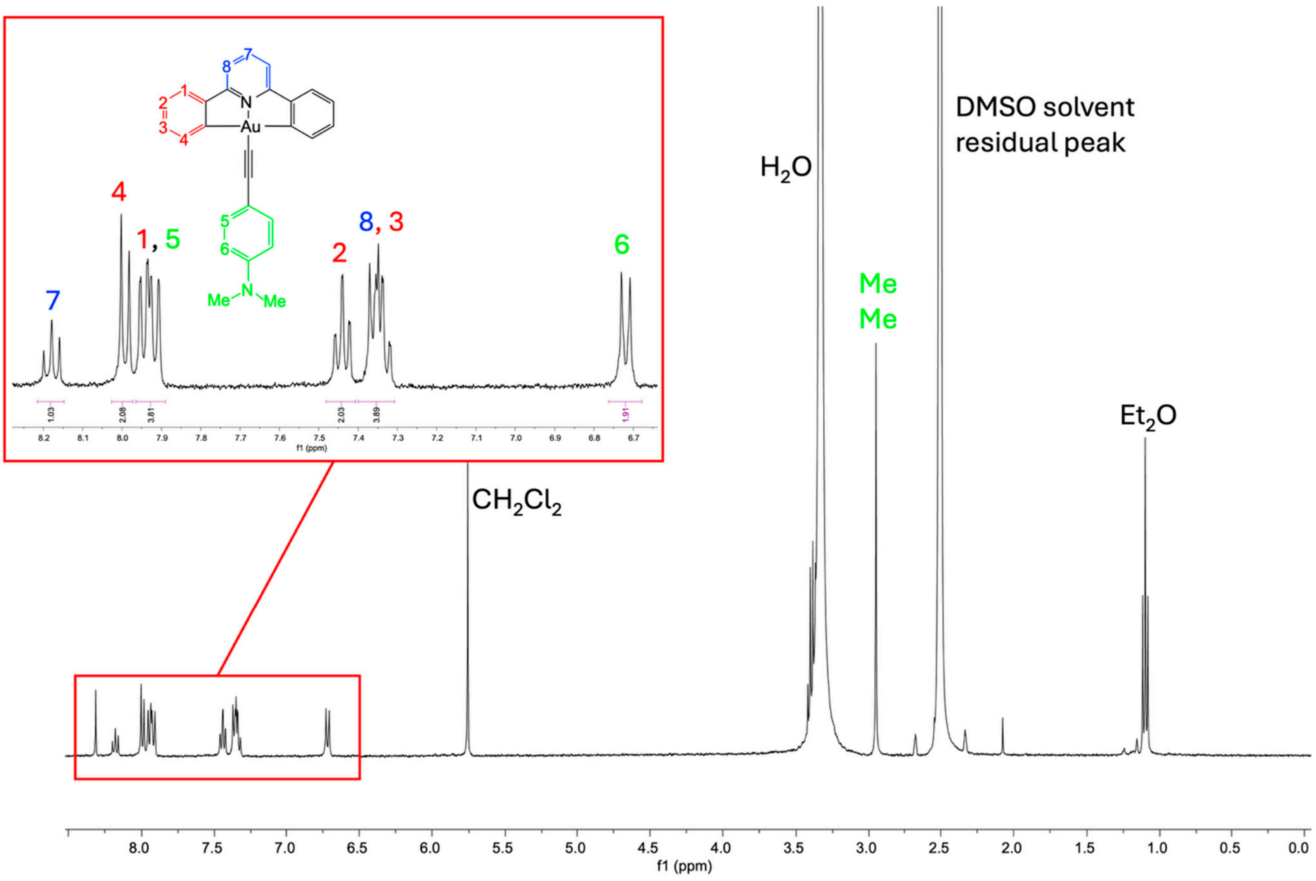

Figure S10. ^{1}H NMR spectrum of **Au-CC-dmal**, 400 MHz, DMSO-d_6, 25°C.

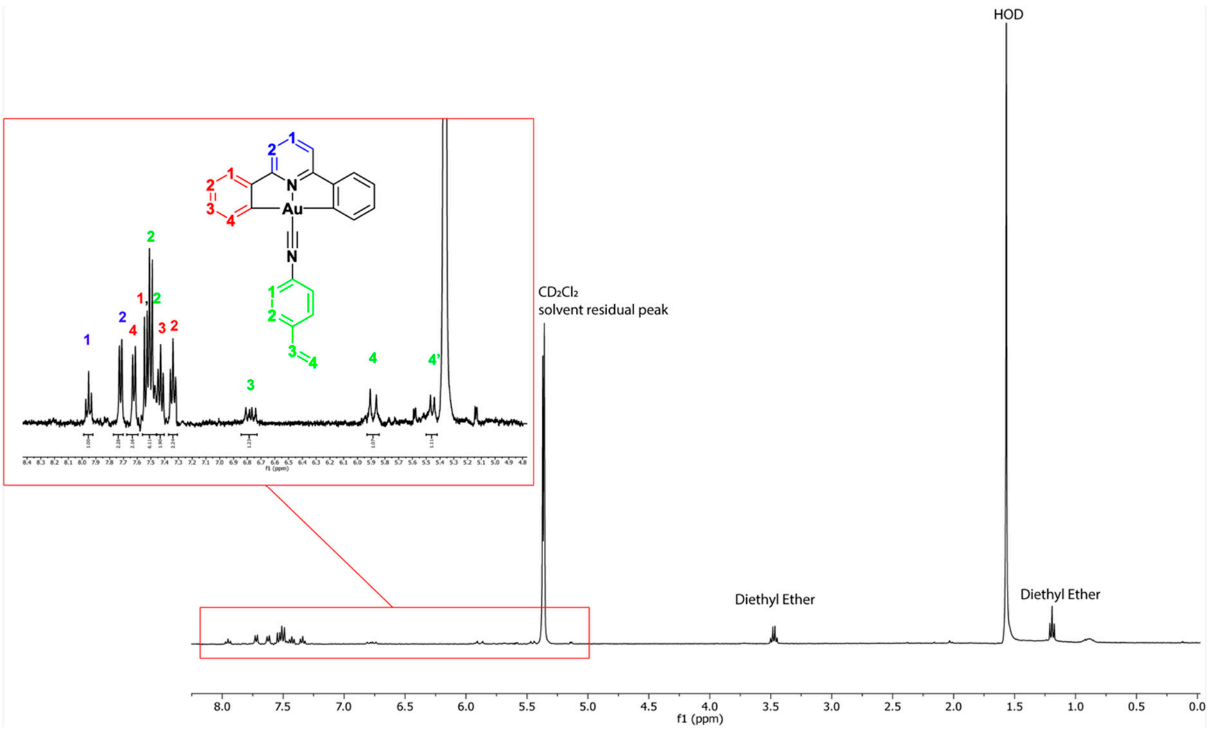

Figure S11. ^{1}H NMR spectrum of **Au-CN-styr**, 400 MHz, CD_2Cl_2, 25ºC.

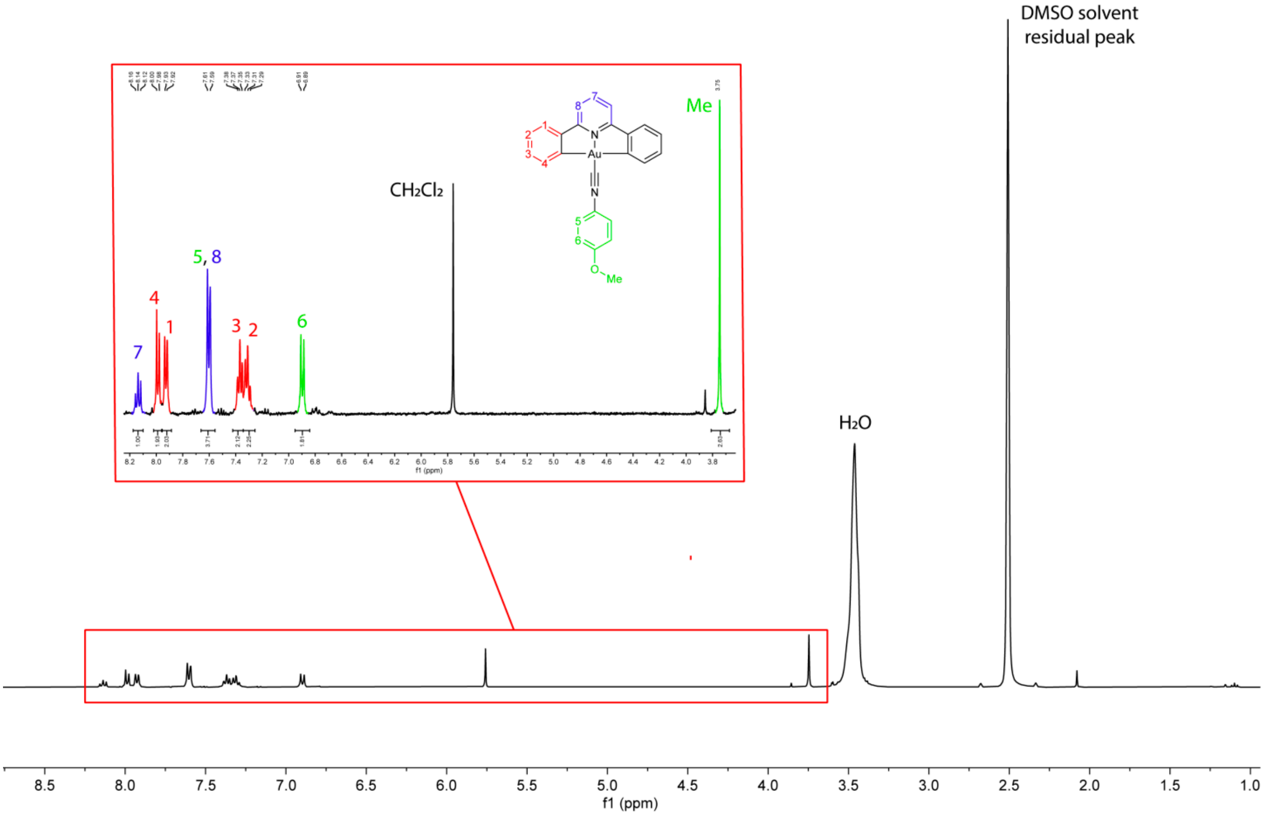

Figure S12. ^{1}H NMR spectrum of **Au-CN-anis**, 400 MHz, DMSO-d_6, 25ºC.

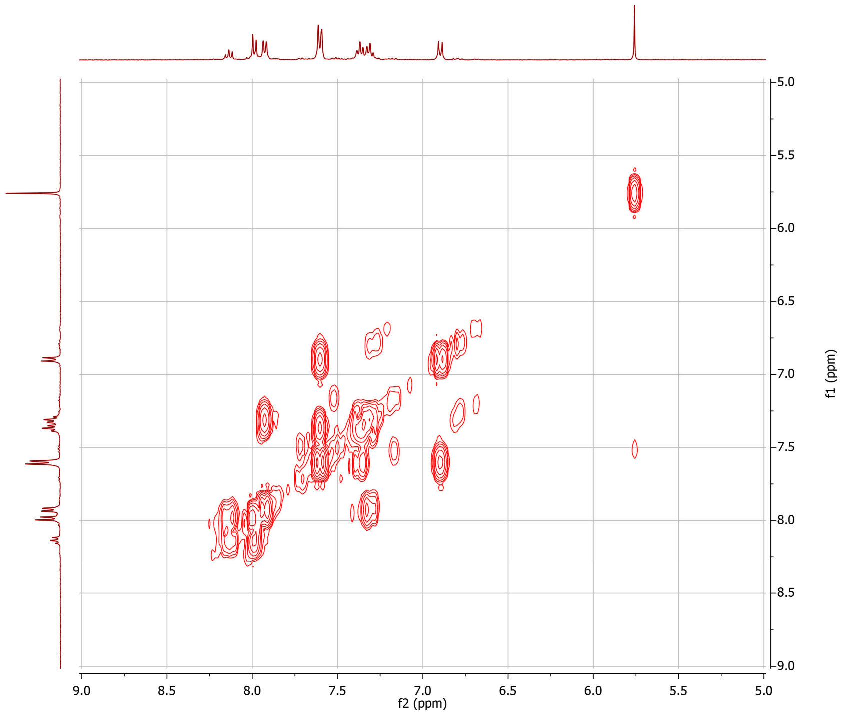

Figure S13. ^{1}H-^{1}H COSY NMR spectrum of **Au-CN-anis**, 400 MHz, DMSO-d$_6$, 25ºC.

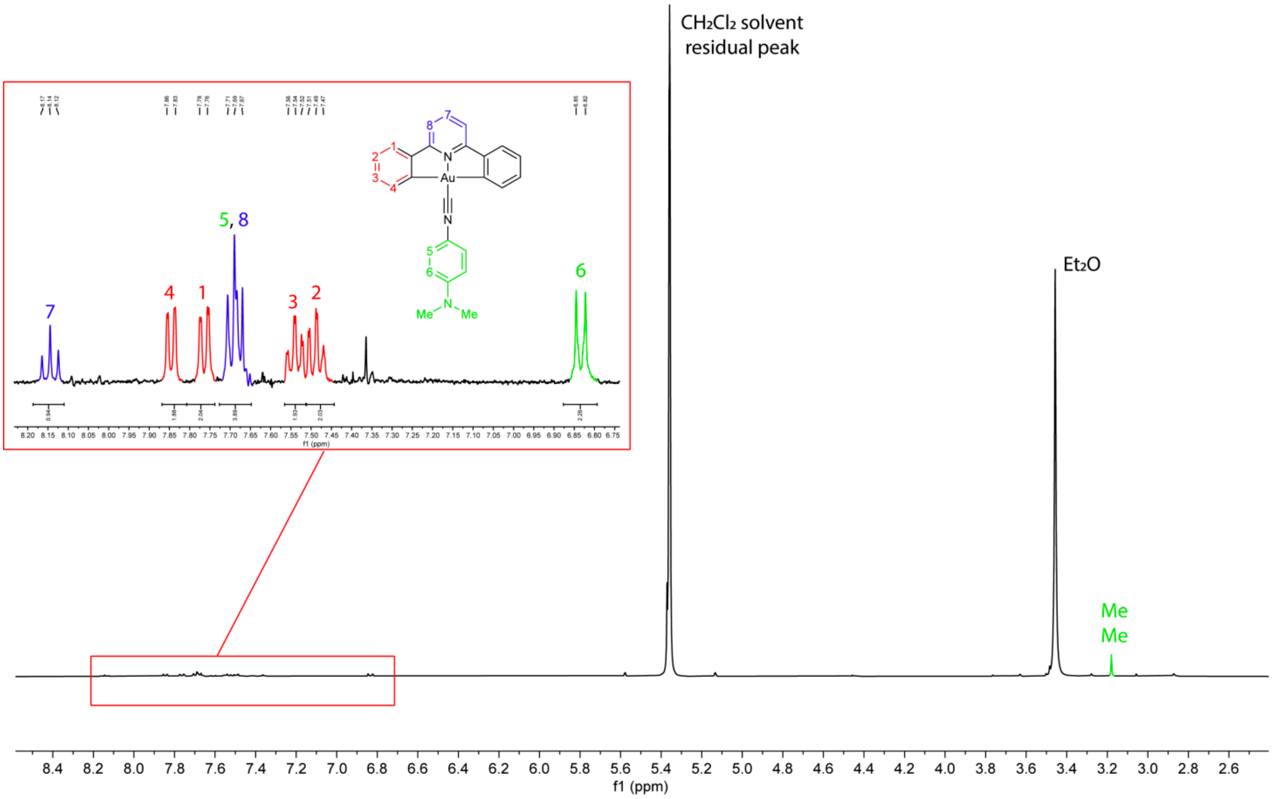

Figure S14. ^{1}H NMR spectrum of **Au-CN-dmal**, 400 MHz, CD_2Cl_2, 25ºC.

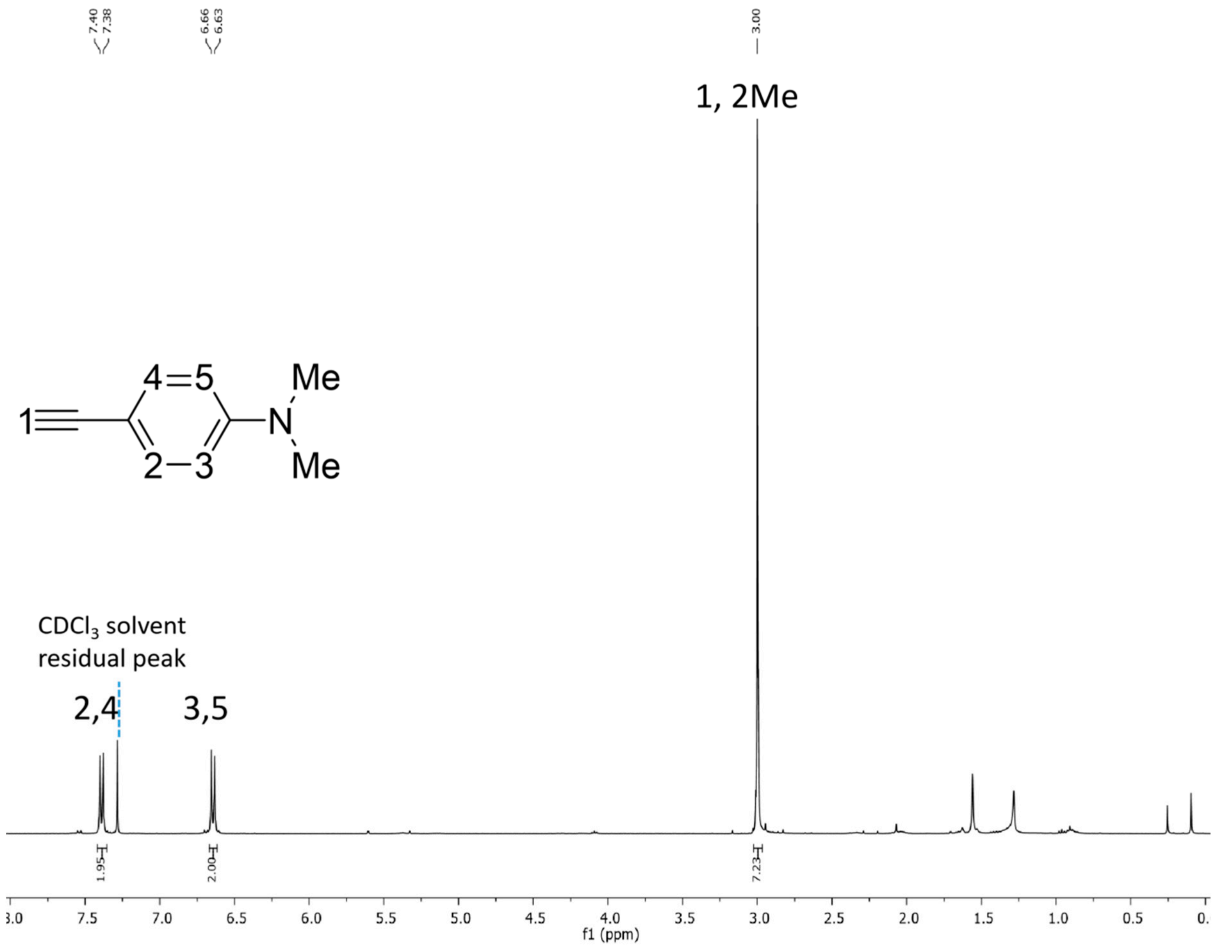

Figure S15. ^{1}H NMR spectrum of **4-Ethynyl-N,N-dimethylaniline**, 400 MHz, $CDCl_3$, 25°C.

S3. Photophysical data

Table S3. Excited-state lifetimes of the **Au-CC-anis**, **Au-CN-dmal**, **Au-CN-anis** in CH_2Cl_2 solution and **Au-CN-anis**, **Au-CC-dmal** in solid state were determined by fitting the emission decays with a biexponential model, together with the relative contribution of each component to the average lifetime.

		A_1	$\tau 1$	A_2	$\tau 2$	x_1	x_2	τ_{av}
**Au-CN-anis**	solid	1582	**42**	75	**194**	0.82	0.18	**69**
	solution	5567	**26**	325	**77**	0.85	0.15	**33**
**Au-CN-dmal**	solution	3621	**34**	86	**142**	0.92	0.08	**44**
**Au-CC-anis**	solution	19781	**336**	1381	**780**	0.86	0.14	**398**
**Au-CC-dmal**	solid	888	**49**	96	**199**	0.71	0.29	**93**

The average lifetime (τ_{av}) and relative contribution of each component (x_i) were determined using the following equations:

$$\tau_{av} = \frac{A_1\tau_1^2 + A_2\tau_2^2}{A_1\tau_1 + A_2\tau_2}$$

$$x_i = \frac{A_i\tau_i}{A_1\tau_1 + A_2\tau_2}$$

- A_1, A_2 – the amplitudes of each component of the decay;
- τ_1, τ_2 – lifetimes of each component;

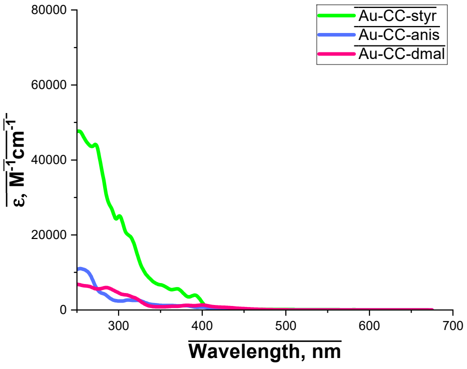

A)

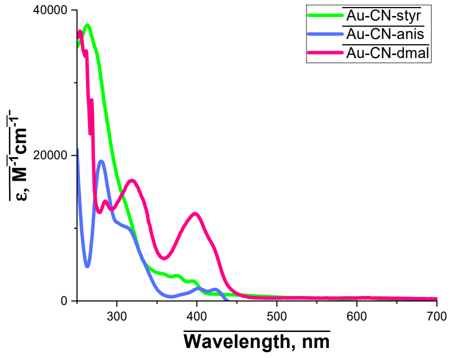

B)

Figure S16. Absorption spectra of Au(III) alkynyl and isocyanide complexes in CH_2Cl_2 solution at concentration 10^{-5} mol/L.

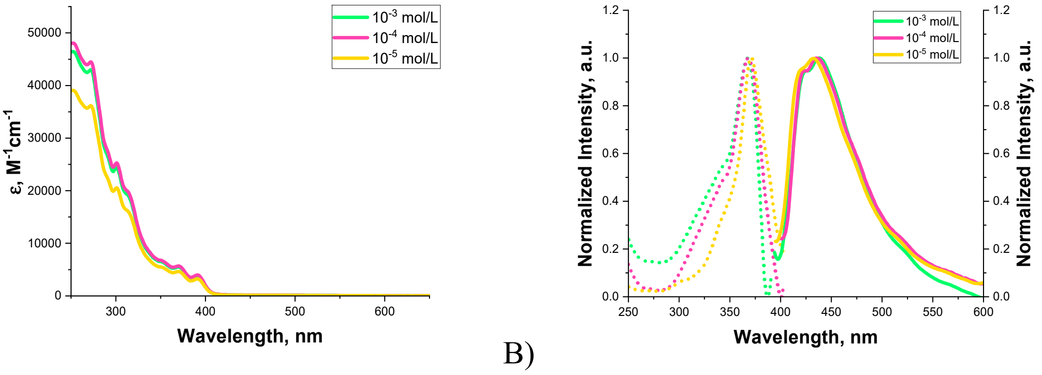

A) B)

Figure S17. Absorption (A), excitation and emission (B) spectra of **Au-CC-styr** in CH_2Cl_2 solutions at concentrations 10^{-3} – 10^{-5} mol/L.

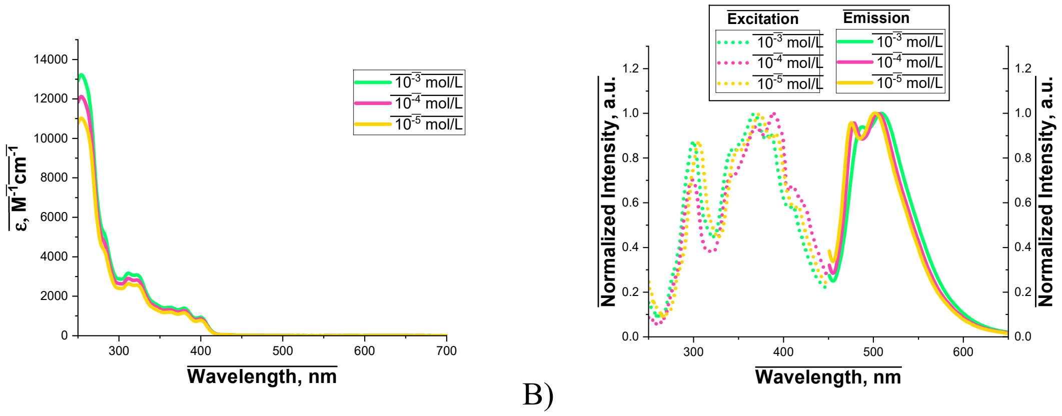

A) B)

Figure S18. Absorption (A), excitation and emission (B) spectra of **Au-CC-anis** in CH_2Cl_2 solutions at concentrations 10^{-3} – 10^{-5} mol/L.

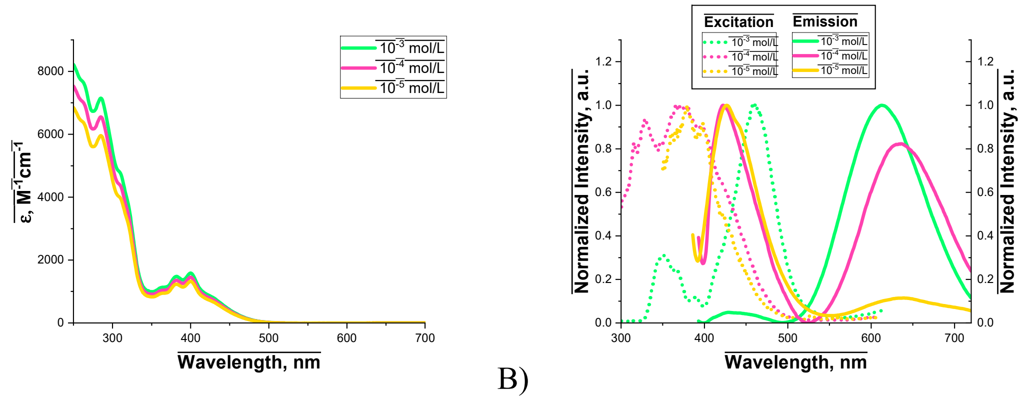

A) B)

Figure S19. Absorption (A), excitation and emission (B) spectra of **Au-CC-dmal** in CH_2Cl_2 solutions at concentrations 10^{-3} – 10^{-5} mol/L.

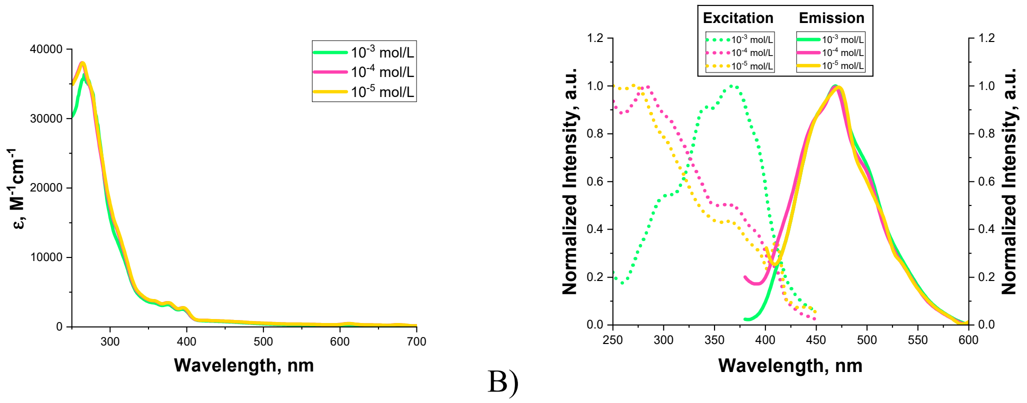

A) B)

Figure S20. Absorption (A), excitation and emission (B) spectra of **Au-CN-styr** in CH_2Cl_2 solutions at concentrations 10^{-3} – 10^{-5} mol/L.

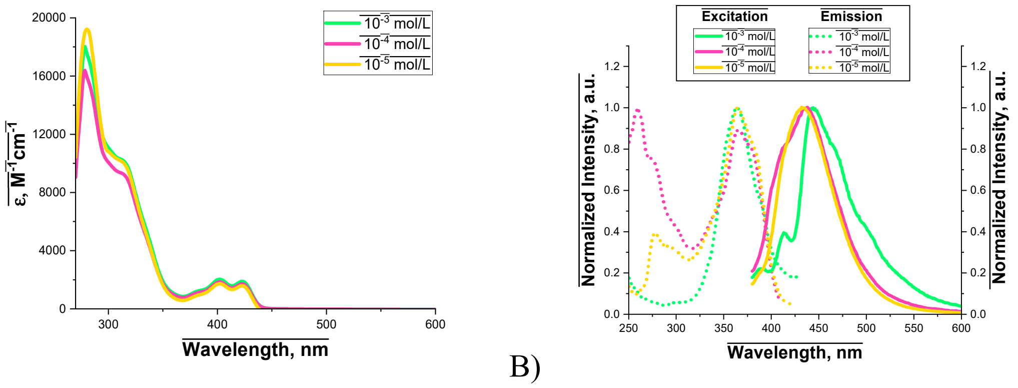

A) B)

Figure S21. Absorption (A), excitation and emission (B) spectra of **Au-CN-anis** in CH_2Cl_2 solutions at concentrations 10^{-3} – 10^{-5} mol/L.

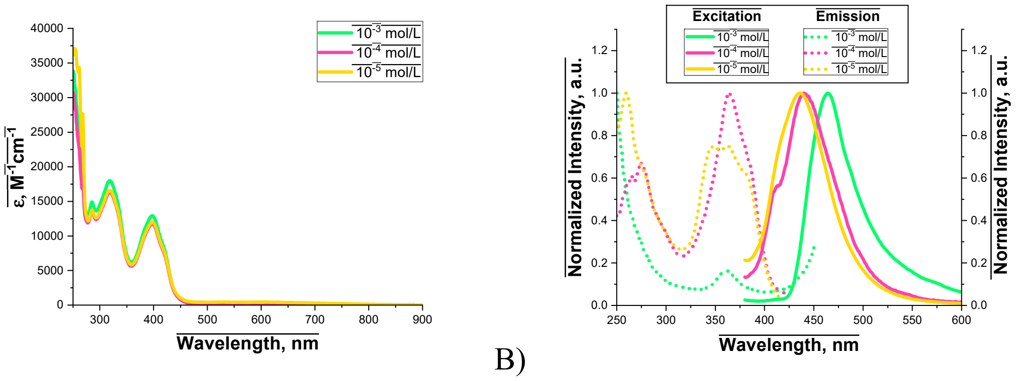

A) B)

Figure S22. Absorption (A), excitation and emission (B) spectra of **Au-CN-dmal** in CH_2Cl_2 solutions at concentrations 10^{-3} – 10^{-5} mol/L.

S4. Calculations

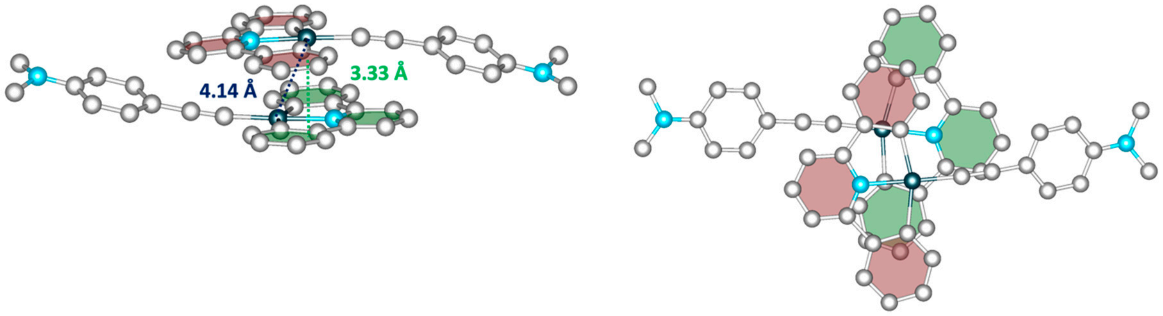

Au-CC-dmal (180º)

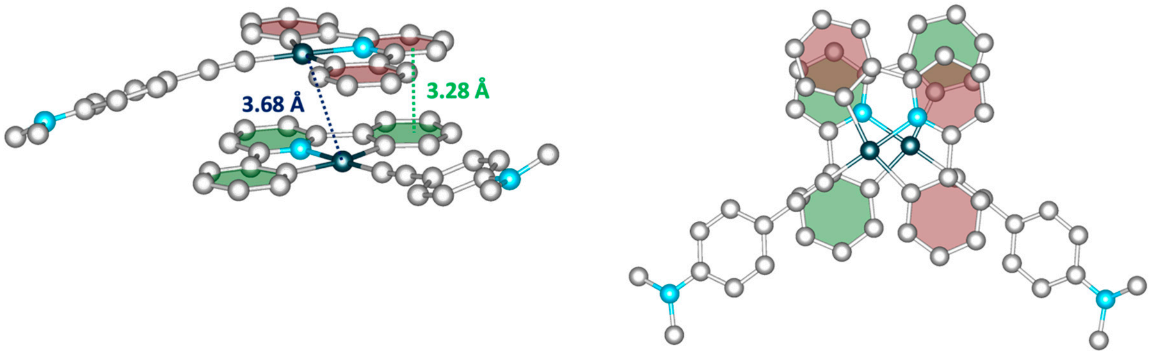

Au-CC-dmal (90º)

Figure S23. Ground state (S_0) optimized structure of **Au-CC-dmal** aggregated dimers.

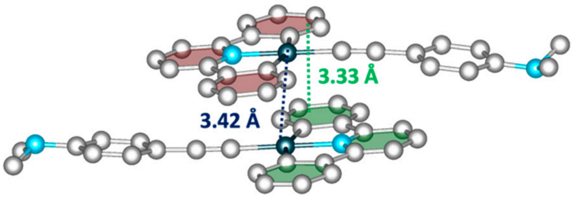

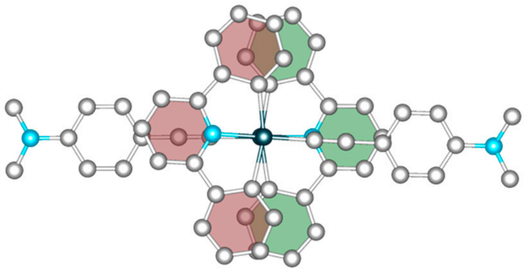

$\lambda_{em} = 487\ nm$

Au-CC-dmal (180º)

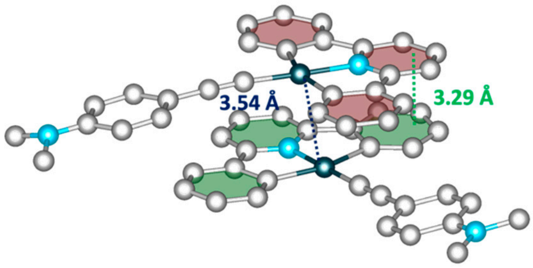

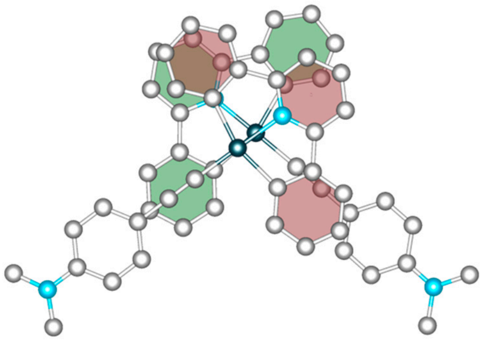

$\lambda_{em} = 513\ nm$

Au-CC-dmal (90º)

Figure S24. Excited state (T_1) optimized structure of **Au-CC-dmal** aggregated dimers.

Table S4. Interfragment charge transfer in the S0 → T1 transition calculated for monomer model of the **Au-CC-dmal** in DCM calculated at the M062X/(6-31g*, Au SDD) (PCM = DCM) level of theory.

**Au-CC-dmal**	**Au**	**C^N^C**	**CC-C6H5**	**N(CH3)2**
**Au**	0.00034			
**C^N^C**	0.0188	0.95908		
**CC-C6H5**	0.00006	0.00312	0	
**N(CH3)2**	0	0	0	0

Table S5. Interfragment charge transfer in the S0 → T1 transition calculated for dimer model of the **Au-CC-dmal-180** in the gas phase calculated at the M062X/(6-31g*, Au SDD) level of theory.

**Au-CC-dmal**	**Au**	**C^N^C**	**CC-C6H5**	**N(CH3)2**	**Au**	**C^N^C**	**CC-C6H5**	**N(CH3)2**

**Au**	0.00008							
**C^N^C**	0.00015	0.00251						
**CC-C6H5**	0.00005	0.00085	0.00021					
**N(CH3)2**	0	0.00001	0	0				
**Au**	0.00043	0.00711	0.00179	0.00008	0.00125			
**C^N^C**	0.00543	0.08994	0.02262	0.00105	0.01582	0.71613		
**CC-C6H5**	0.00017	0.00279	0.0007	0.00003	0.00049	0.02219	0.00022	
**N(CH3)2**	0.00001	0.00012	0.00003	0	0.00002	0.00095	0.00001	0

Table S6. Interfragment charge transfer in the S0 → T1 transition calculated for dimer model of the **Au-CC-dmal-90** in DCM calculated at the M062X/(6-31g*, Au SDD) (PCM = DCM) level of theory.

**Au-CC-dmal-90**	**Au**	**C^N^C**	**CC-C6H5**	**N(CH3)2**	**Au**	**C^N^C**	**CC-C6H5**	**N(CH3)2**
**Au**	0.00006							
**C^N^C**	0.00013	0.00104						
**CC-C6H5**	0.00001	0.00029	0.00001					
**N(CH3)2**	0	0.00001	0	0				
**Au**	0.00025	0.00415	0.0001	0.00003	0.00546			
**C^N^C**	0.00221	0.00666	0.00015	0.00005	0.00876	0.00307		
**CC-C6H5**	0.00009	0.00817	0.00019	0.00006	0.01075	0.00376	0.26862	
**N(CH3)2**	0.00001	0.00094	0.00002	0.00001	0.00123	0.00043	0.03074	0.01085

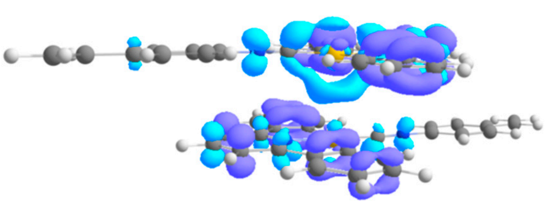

$\lambda_{em} = 528\, nm$

Au-CN-styr

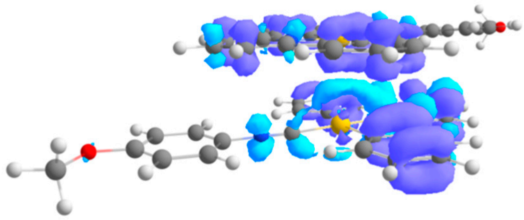

$\lambda_{em} = 534\, nm$

Au-CN-anis

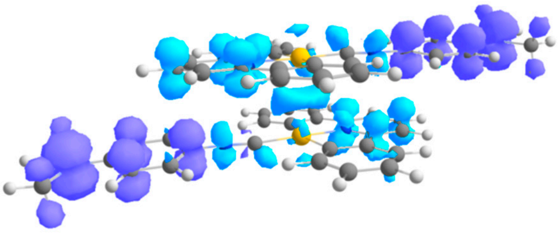

$\lambda_{em} = 553\, nm$

Au-CN-dmal

Figure S25. The decrease (violet) and increase (cyan) of electron density for the S0 → T1 transition calculated for dimer models of **Au-CN-styr**, **Au-CN-anis**, **Au-CN-dmal** complexes in the gas phase at the MN12SX/(6-31g*, Au SDD) level of theory. The calculated emission maxima are given under the corresponding structures.

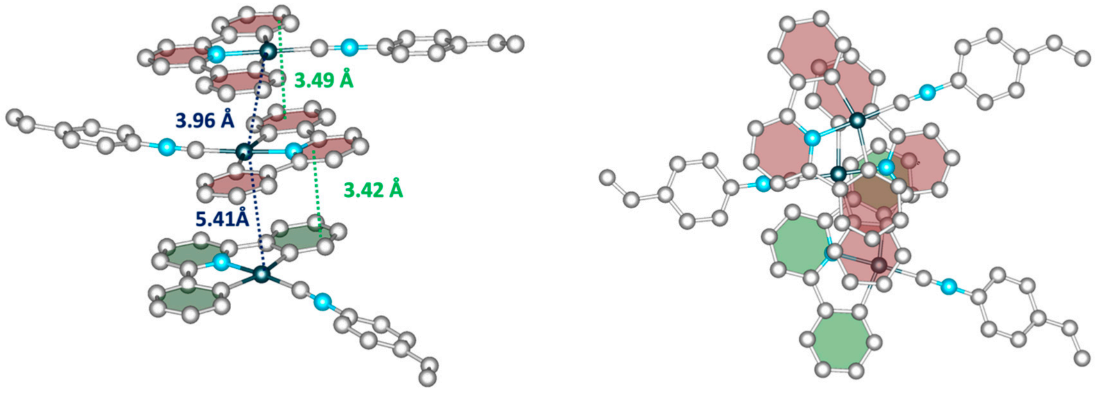

Au-CN-styr

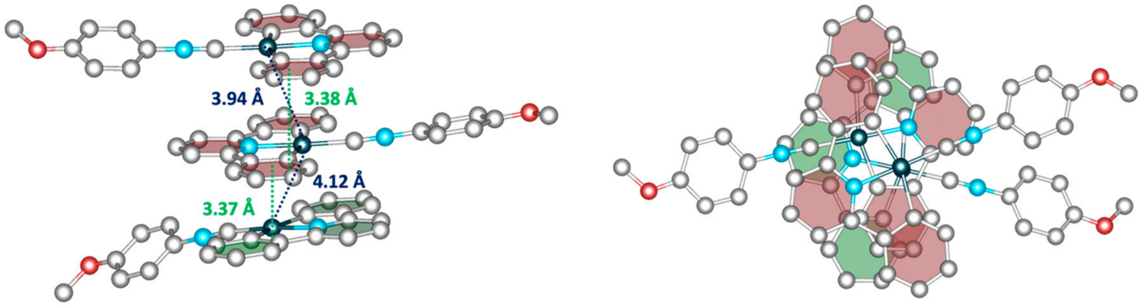

Au-CN-anis

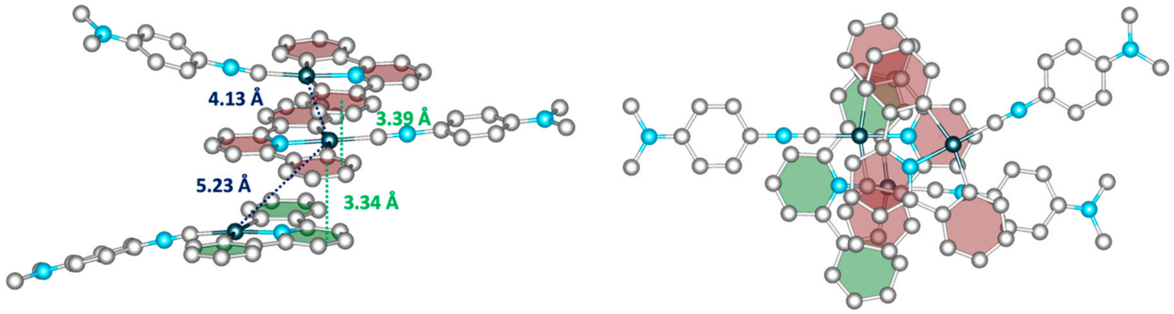

Au-CN-dmal

Figure S26. Ground state (S_0) optimized structures of aggregated trimers for isocyanide complexes.

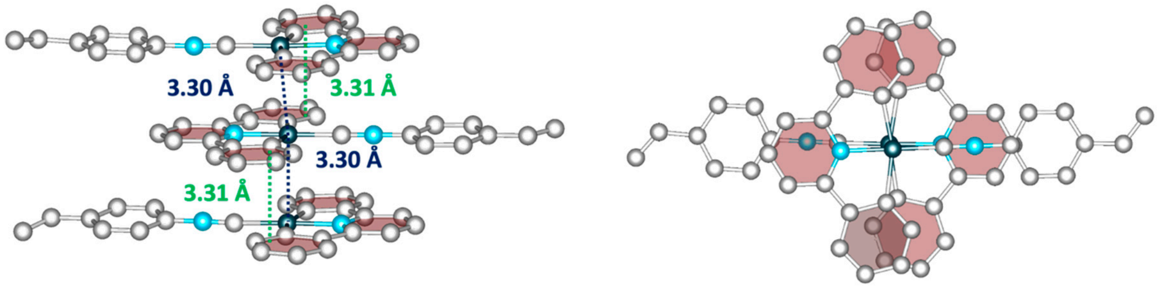

Au-CN-styr

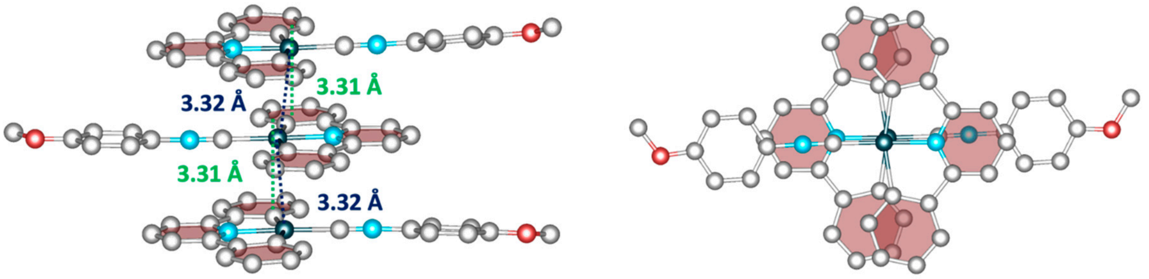

Au-CN-anis

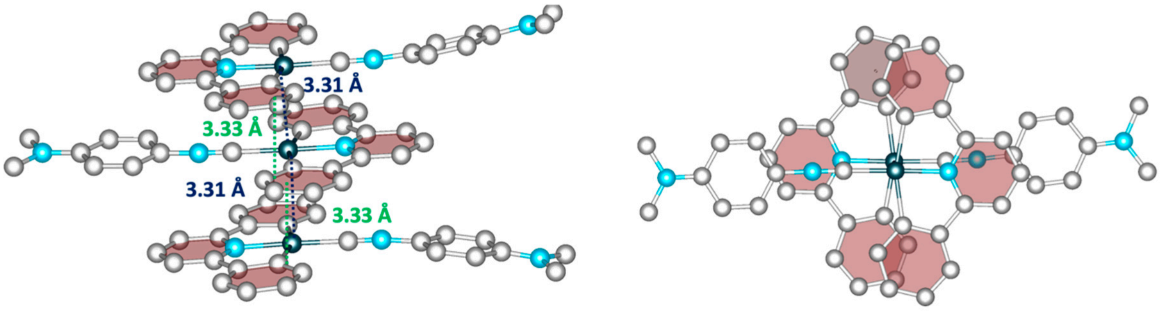

Au-CN-dmal

Figure S27. Excited T_1 state optimized structures of aggregated trimers for isocyanide complexes.

Table S7. Interfragment charge transfer in the S0 → T1 transition calculated for monomer model of the **Au-CN-styr** in the gas phase calculated at the MN12SX/(6-31g*, Au SDD) (PCM = DCM) level of theory.

Au-CN-styr	Au	C^N^C	CN-C6H5	C2H5
**Au**	0.00262			
**C^N^C**	0.01709	0.79024		
**CN-C6H5**	0.0014	0.06484	0.00001	
**C2H5**	0.00006	0.0026	0	0

Table S8. Interfragment charge transfer in the S0 → T1 transition calculated for monomer model of the **Au-CN-anis** in the gas phase calculated at the MN12SX/(6-31g*, Au SDD) (PCM = DCM) level of theory.

**Au-CN-anis**	**Au**	**C^N^C**	**CN-C6H5**	**OCH3**
**Au**	0.00262			
**C^N^C**	0.01679	0.77735		
**CN-C6H5**	0.00181	0.08394	0.00002	
**OCH3**	0.00007	0.00304	0	0

Table S9. Interfragment charge transfer in the S0 → T1 transition calculated for monomer model of the **Au-CN-dmal** in the gas phase calculated at the MN12SX/(6-31g*, Au SDD) (PCM = DCM) level of theory.

**Au-CN-dmal**	**Au**	**C^N^C**	**CN-C6H5**	**N(CH3)2**
**Au**	0.00134			
**C^N^C**	0.00622	0.01536		
**CN-C6H5**	0.0007	0.00172	0.04745	
**N(CH3)2**	0	0.00001	0.00016	0.00011

Table S10. Interfragment charge transfer in the S0 → T1 transition calculated for dimer model of the **Au-CN-styr** in the gas phase calculated at the MN12SX/(6-31g*, Au SDD) level of theory.

**Au-CN-styr**	**Au**	**C^N^C**	**CN-C6H5**	**C2H5**	**Au**	**C^N^C**	**CN-C6H5**	**C2H5**
**Au**	0.00196							
**C^N^C**	0.00473	0.1814						
**CN-C6H5**	0.00194	0.07416	0.00032					
**C2H5**	0.00017	0.00647	0.00003	0				
**Au**	0.00169	0.06494	0.00028	0	0.00099			

**C^N^C**	0.00368	0.14105	0.0006	0	0.00214	0.07662		
**CN-C6H5**	0.00169	0.06477	0.00028	0	0.00098	0.03518	0.00171	
**C2H5**	0.00015	0.00568	0.00002	0	0.00009	0.00309	0.00015	0.00008

Table S11. Interfragment charge transfer in the S0 → T1 transition calculated for dimer model of the **Au-CN-anis** in the gas phase calculated at the MN12SX/(6-31g*, Au SDD) level of theory.

**Au-CN-anis**	**Au**	**C^N^C**	**CN-C6H5**	**OCH3**	**Au**	**C^N^C**	**CN-C6H5**	**OCH3**
**Au**	0.00240							
**C^N^C**	0.00077	0.00580						
**CN-C6H5**	0.00021	0.00159	0.000					
**OCH3**	0.00009	0.00067	0.000	0.0				
**Au**	0.00123	0.00922	0.00002	0.0	0.00065			
**C^N^C**	0.00006	0.00048	0.000	0.0	0.00003	0.00009		
**CN-C6H5**	0.00007	0.00049	0.000	0.0	0.00003	0.00009	0.00001	
**OCH3**	0.00004	0.00027	0.000	0.0	0.00002	0.00005	0.00001	0.00001

Table S12. Interfragment charge transfer in the S0 → T1 transition calculated for dimer model of the **Au-CN-dmal** in the gas phase calculated at the MN12SX/(6-31g*, Au SDD) level of theory.

**Au-CN-dmal**	**Au**	**C^N^C**	**CN-C6H5**	**N(CH3)2**	**Au**	**C^N^C**	**CN-C6H5**	**N(CH3)2**
**Au**	0.00073							
**C^N^C**	0.00007	0.00001						
**CN-C6H5**	0.00005	0.00000	0.00001					

**N(CH3)2**	0.00008	0.00001	0.00002	0.00234				
**Au**	0.00074	0.00005	0.00020	0.02280	0.00077			
**C^N^C**	0.00007	0.00001	0.00002	0.00214	0.00007	0.00000		
**CN-C6H5**	0.00005	0.00	0.00001	0.00160	0.00005	0.0000	0.00001	
**N(CH3)2**	0.00006	0.00	0.00002	0.00192	0.00006	0.0000	0.00002	0.00168

Table S13. Interfragment charge transfer in the S0 → T1 transition calculated for trimer model of the **Au-CN-styr** in the gas phase calculated at the MN12SX/(6-31g*, Au SDD) level of theory.

**Au-CN-styr**	**Au**	**C^N^C**	**CN-C6H5**	**C2H3**	**Au**	**C^N^C**	**CN-C6H5**	**C2H3**	**Au**	**C^N^C**	**CN-C6H5**	**C2H3**
**Au**	0.00055											
**C^N^C**	0.00059	0.01887										
**CN-C6H5**	0.00024	0.00765	0.00038									
**C2H3**	0.00002	0.00054	0.00003	0.00002								
**Au**	0.0007	0.02221	0.00109	0.00072	0.00086							
**C^N^C**	0.0031	0.0984	0.00482	0.00318	0.00379	0.17196						
**CN-C6H5**	0.00104	0.0331	0.00162	0.00107	0.00127	0.05785	0.0005					
**C2H3**	0.00009	0.00285	0.00014	0.00009	0.00011	0.00498	0.00004	0.00001				
**Au**	0.00054	0.01722	0.00084	0.00056	0.00066	0.0301	0.00026	0.00004	0.00059			
**C^N^C**	0.00061	0.01944	0.00095	0.00063	0.00075	0.03397	0.00029	0.00005	0.00067	0.01835		
**CN-C6H5**	0.00023	0.00736	0.00036	0.00024	0.00028	0.01286	0.00011	0.00002	0.00025	0.00694	0.0002	
**C2H3**	0.00002	0.00048	0.00002	0.00002	0.00002	0.00084	0.00001	0	0.00002	0.00045	0.00001	0.00001

Table S14. Interfragment charge transfer in the S0 → T1 transition calculated for trimer model of the **Au-CN-anis** in the gas phase calculated at the MN12SX/(6-31g*, Au SDD) level of theory.

**Au-CN-anis**	**Au**	**C^N^C**	**CN-C6H5**	**OCH3**	**Au**	**C^N^C**	**CN-C6H5**	**OCH3**	**Au**	**C^N^C**	**CN-C6H5**	**OCH3**

**Au**	0.00055											
**C^N^C**	0.00056	0.01633										
**CN-C6H5**	0.00015	0.00437	0.00003									
**OCH3**	0	0.00013	0	0								
**Au**	0.00088	0.02571	0.00018	0.00002	0.00094							
**C^N^C**	0.00413	0.12032	0.00086	0.00011	0.00442	0.15829						
**CN-C6H5**	0.00099	0.02892	0.00021	0.00003	0.00106	0.03804	0.00023					
**OCH3**	0.00004	0.0013	0.00001	0	0.00005	0.0017	0.00001	0				
**Au**	0.00067	0.01967	0.00014	0.00002	0.00072	0.02587	0.00016	0	0.00053			
**C^N^C**	0.00085	0.02489	0.00018	0.00002	0.00091	0.03274	0.0002	0	0.00067	0.02151		
**CN-C6H5**	0.00028	0.00802	0.00006	0.00001	0.00029	0.01055	0.00006	0	0.00022	0.00693	0.00009	
**OCH3**	0.00001	0.00029	0	0	0.00001	0.00038	0	0	0.00001	0.00025	0	0

Table S15. Interfragment charge transfer in the S0 → T1 transition calculated for trimer model of the **Au-CN-dmal** in the gas phase calculated at the MN12SX/(6-31g*, Au SDD) level of theory.

**Au-CN-dmal**	**Au**	**C^N^C**	**CN-C6H5**	**N(CH3)2**	**Au**	**C^N^C**	**CN-C6H5**	**N(CH3)2**	**Au**	**C^N^C**	**CN-C6H5**	**N(CH3)2**
**Au**	0.00051											
**C^N^C**	0.00065	0.00044										
**CN-C6H5**	0.00017	0.00011	0.00828									
**N(CH3)2**	0.00002	0.00001	0.00089	0.00077								
**Au**	0.00067	0.00046	0.03351	0.02894	0.00002							
**C^N^C**	0.00358	0.00244	0.17882	0.15446	0.00008	0.00319						
**CN-C6H5**	0.00081	0.00055	0.04025	0.03477	0.00002	0.00072	0.00001					
**N(CH3)2**	0.00011	0.00007	0.00532	0.0046	0	0.00009	0	0				
**Au**	0.0006	0.00041	0.02977	0.02572	0.00001	0.00053	0.00001	0	0.00017			
**C^N^C**	0.0009	0.00061	0.0449	0.03878	0.00002	0.0008	0.00001	0	0.00026	0.0002		
**CN-C6H5**	0.00032	0.00022	0.01606	0.01387	0.00001	0.00029	0	0	0.00009	0.00007	0.00388	
**N(CH3)2**	0.00004	0.00003	0.00188	0.00162	0	0.00003	0	0	0.00001	0.00001	0.00045	0.00041
